# Matching different-structured advertising pictorial metaphors with verbalization forms: incongruity-based evoked response potentials evidence

**DOI:** 10.3389/fpsyg.2023.1131387

**Published:** 2023-05-16

**Authors:** Shuo Cao, Fang Yue, Shihui Zheng, Yang Fu, Jing Huang, Huili Wang

**Affiliations:** ^1^School of Foreign Languages, Dalian University of Technology, Dalian, China; ^2^Faculty of Management and Economics, Dalian University of Technology, Dalian, China; ^3^Instituto de Neurociencia IUNE, Facultad de Psicología, Universidad de La Laguna, San Cristóbal de La Laguna, Spain; ^4^School of Foreign Languages, Hangzhou City University, Hangzhou, China

**Keywords:** pictorial metaphors, visual structure, verbalization, incongruity, ERP

## Abstract

Many studies emphasize the need of verbally representing pictorial metaphors, but few have empirically investigated whether and how the particular verbalization form match different types of pictorial metaphors. Using evoked response potentials (ERP), a 3 (pictorial structure: fusion, juxtaposition, literal image) × 2 [verbalization form: A是(is) B, A像(is like) B] within-group experiment was conducted among 36 participants. ERPs were time-locked to the onset of the verb [是/像(is/is like)] of the metaphor sentence that follows a pictorial metaphor to detect the verbo-pictorial incongruity in metaphor comprehension. The incongruity-based ERP analysis showed that pictorial metaphors, when verbalized in two forms, all induced frontal N1 effect, regardless of pictorial structures, only with a larger N1 amplitude for literal images in “A是(is) B.” A central stronger P2 was observed in “A像(is like) B” for three structures. Despite a general elicitation of posterior P3 in all conditions, a larger P3 was found for juxtapositions verbalized in “A像(is like) B” and for literal images verbalized in “A是(is) B.” There was no significant difference between two verbalization forms for fusion-structured pictorial metaphors. These findings suggest: (1) verbo-pictorial metaphors could induce incongruity-based attention; (2) higher verbo-pictorial semantic congruity and relatedness, indexed by stronger P2 and P3, confirmed “A像(is like) B” to be the more effective verbalization form in representing pictorial metaphors, specifically for juxtaposition-structured pictorial metaphors; (3) for non-metaphor advertising pictures, verbal metaphor showed an interference effect. The study not only reveals the neuro-cognitive mechanism of processing verbo-pictorial metaphors, but also offers neural reference for the design of effective multi-modal metaphor by finding an optimal match between PMs and verbalization forms.

## 1. Introduction

Visual metaphors are visual manifestations of cognitive metaphors where concepts are represented in images (Lakoff and Johnson, 2008). As an indirect persuasion popular in advertising, pictorial metaphors (hereafter as PM) imply advertising appeals in an implicit manner, as a PM usually combines two dissimilar objects for viewers to discover the subtle connections. In the PM comprehension, the contextual verbal clues play an important role (Phillips and McQuarrie, 2004; Forceville and Urios-Aparisi, 2009; Bergkvist et al., 2012).

On the cognitive processing of verbo-pictorial metaphors, visual structures of PM, concerned with the visual position and relationship of one thing to another, have an important influence. Considering differed processing complexity, a few typologies to the classification of visual metaphors were proposed (Forceville, 1996; Phillips and McQuarrie, 2004; Gkiouzepas and Hogg, 2011; Peterson, 2019). Typically, Phillips and McQuarrie (2004) classification includes fusion, juxtaposition and replacement. This typology, consistent with Forceville’ classification (hybrid metaphor, pictorial simile and context metaphor), has been widely used in empirical metaphor studies (Indurkhya and Ojha, 2013; van Mulken et al., 2014; Ojha et al., 2017; Ryoo et al., 2021). For the three structures, a gradient of complexity was previously presupposed and confirmed, with the lowest for juxtaposition and the highest for replacement. Fusions are graded as of moderate complexity. In a juxtaposition-structure PM, the source and target are visually equated, being presented side by side in a juxtaposition. As for fusion-structure, the source and target are combined to merge into one, so that viewers need to make some efforts to break down the two elements for comprehending the meaning intended by the PM. The most complex one is replacement-structure that has one domain absent. Viewers need to identify the missing element based on the information indicated by the present domain, verbal messages and context of the advertisement, which consumes excessive processing effort and usually exceeds cognitive resources that viewers have available (McQuarrie and Mick, 1999; Brennan and Bahn, 2006).

In addition to the visual structure of PMs, the effect study of verbalized manifestation on PM comprehension is also determined by the necessity of translating PMs into language. As put by Ojha et al. (2017), comprehension of visual metaphors required the involvement of language resources and in many cases, verbalization is necessary. In practice, PMs are also often presented with verbal messages in the headline to illustrate PMs (Mothersbaugh et al., 2002). Researchers and advertising designers all believe that metaphors, whether verbal or visual, are primarily multi-modal, which requires the allocation and integration of information from different modalities to resolve the inter-domain incongruity created by a metaphor to make sense of its implied meaning. In a multi-modal metaphor, pictorial and verbal elements interact in a dynamic way (Leigh, 1994; McQuarrie and Phillips, 2005; van Mulken et al., 2014). Different from verbal metaphors, in PM there are not such linearity or grammatical rules as the explicit copula “is” or “like,” which renders difficulty for viewers in distinguishing target domains from source domains due to the uncertainty of conceptual mapping (Forceville, 2008). Some studies explored the positive effect of verbal elements on the PM comprehension (Phillips, 2000; Ang and Lim, 2006; Bergkvist et al., 2012; Lagerwerf et al., 2012). When verbal elements were added to PMs, it would be easier for PMs to be understood because the verbal cues were provided to reduce the required processing complexity for ad comprehension by strengthening the connections between two visual elements in PMs (Lagerwerf et al., 2012). Phillips (2000) proposed that an implicit headline was desirable to increase the level of comprehension of the advertising metaphors, which in turn elicited a positive effect on ad liking. Whereas an explicit headline produced increased comprehension but decreased ad liking by reducing consumers’ pleasure in interpreting the message of the ad. As Ang and Lim (2006) claimed, the effect of verbal metaphors is dynamic, depending on product type (symbolic products vs. utilitarian products), picture type (metaphor vs. non-metaphor) and headline type (metaphor vs. non-metaphor). As such, due to the complicated and divergent findings, verbalized PM calls for a further study.

Based on the previous studies, verbalization of PMs can be realized by several syntactic forms. Forceville (1996) maintained that different forms of verbalization reflect different ways we experience metaphors. Despite various grammatical structures for metaphorical sentences, Lakoff and Johnson (2008) suggested that all metaphorical expressions derive from the conceptual paradigmatic form “A is B.” This combination of linearity and syntactic rules allows for an easy distinguishing between the target domain and the source domain and for a clear identification of the features mapped from source to target. However, the career of metaphor theory (Bowdle and Gentner, 2005) proposed different syntactic structures (“A is B” and “A is like B”) for the manifestation of metaphorical thinking via an empirical study that investigated the preferred processing route of verbal metaphors. They designed their experiment based on what is known as grammatical concordance (Gibb and Wales, 1990; Gregory and Mergler, 1990), believing that there is a link between form and function in figurative language and that the form follows function. Specifically, the metaphor form (A is B) leads to categorizing the target domain as a member of a specific category named by the source domain so that one can understand one concept in terms of another; in contrast, the simile form (A is like B) facilitates comparing the target domain with the source domain. Therefore, metaphor processing is modulated by conventionality, that is, the form “A is B” fits conventional metaphors best, which uses a categorization-based processing, whereas “A is like B” fits novel metaphors best, which adopts a comparison-based processing. As such, it is clear again that different verbal metaphors are represented in different syntactic forms and recruits different processing mechanisms. Following the similar research stream, Forceville (1996) analyzed linguistic representation of pictorial metaphors in *Pictorial Metaphor in Advertising*, confirming the hypothesis that different-structured PMs can be verbalized by different syntactic forms. He identified the verbalization form “A is B” for MP1s (replacement-structured PM) and MP2s (fusion-structured PM), and the form “A is like B” for PM (juxtaposition-structured PM). Teng and Sun (2002) also contended that visual construction of juxtaposition visually expressed the comparison relation (i.e., A is like B), whereas visual constructions of fusion and replacement visually represented the metaphorical categorization relations (i.e., A is B). These initiative studies laid a solid foundation for the present study in terms of verbalization classification of PMs and of the relationship between verbalization form and corresponding processing mechanism.

In terms of methodology, to the best of our knowledge, experiments using evoked response potential remain less to explore the specific neural mechanism underlying the processing of verbo-pictorial metaphors. Compared with behavioral experiments, the ERP technique can measure cognitive processing without behavioral response in real time. Human’s mental activity originates from the brain, and every change of mental state has a corresponding brain cell activity, which reflects the electrophysiological changes of the cognitive task in the brain. Therefore, due to its high temporal resolution, ERP studies offer a possibility to uncover the time-course of cognitive mechanism underlying PM comprehension. When a picture violates a semantic context, the event-related potential (ERP) is negative compared with that evoked by a picture that fits into the context (Barrett and Rugg, 1990; Pratarelli, 1994; McPherson and Holcomb, 1999). That is, when the picture does not match with the sentence that intends to verbalize it, some ERP components indicating incongruity will be immediately detected. And we can analyze this incongruity and its extent to find the effective verbalization form for PMs.

In the neurocognitive studies of metaphors, ERP components, such as N400 and P600, have been widely investigated for verbal stimuli (Kutas and Hillyard, 1980). It is believed that they reflect different stages of cognitive processing of metaphor comprehension, with N400 reflecting the detection of semantic incongruity (Pynte et al., 1996; Balconi and Amenta, 2010; Kutas and Federmeier, 2011), and P600 marking the late concept matching and semantic integration processing (Van Herten et al., 2005). In addition, early components of ERP, which mainly refer to the potential changes within 200 ms of stimulus onset, can be observed especially when the visual pathway is involved. In the present study, we would focus on some early ERP components, including N1, P2 and P3, so as to discover their initial functional contributions to PM comprehension.

The incongruity-sensitive visual N1 is elicited by visual stimuli and significantly affected by attention (Vogel and Luck, 2000). As an index of a discrimination process within the focus of attention, it is related to filter mechanisms involved in triggering of attention (Boutros et al., 2004; Kisley et al., 2004). In the present experiment, different-structured PMs would attract the attention of participants to varied extent. Accordingly, N1 would be detected in the initial processing stage.

P200, an early component related to visual-spatial processing (O’Donnell et al., 1997; Song et al., 2007; Niu et al., 2008; Tlauka et al., 2009), is reported to be influenced by the contextual predictability, i.e., the efficient semantic features are pre-activated and extracted more rapidly from expected items than from unexpected items. And this contextual predictability could be interpreted as the congruity of the preceding materials with the following stimuli. The more consistent the semantic information, the larger the P2 amplitude. In an experiment by Federmeier and Kutas (2002) who compared sentences ended with contextually expected pictures and those with contextually unexpected pictures. They found larger P2 amplitude in response to expected items. As such, it is hypothesized in the present study that P2 should be detected when the verbo-pictorial matching process is perceived as congruent.

P3, another marker of (in)congruity processing, is found to be associated with a series of cognitive activities in terms of its amplitude, latency, and scalp topography (Pritchard, 1981; Picton, 1992). P3 could be elicited in experimental paradigms, like S1–S2 paradigm (Ma et al., 2008). Changes in P3 are influenced by factors like attention (Mangun and Hillyard, 1990), memory load (Brookhuis et al., 1981), and familiarity of stimuli (Rugg and Doyle, 1992). Ma et al. (2008) proved that P3 can be evoked by category similarity in their experiment of brand extension, in which participants were instructed to decide the suitability between the original brand in S1 and the extension product in S2. The higher similarity and coherence between the brand and the product led to an activation of more neural resources in the brain cortex with a larger P3 amplitude evoked. Therefore, P3 reflects the similarity-based categorical processing.

P3 is widely used to examine similarity (versus incongruity). For example, in an experiment of schematic face conducted by Azizian et al. (2006), stimuli higher in perceptual similarity to targets produced larger P3 than did other stimuli with few similar features. This experiment demonstrated that the P3 amplitude was an effective neural indicator of perceptual similarity between target and non-target stimuli. Watson et al. (2005) further examined the effect of conceptual similarity on the P3 amplitude through an oddball paradigm composed of print words and their conceptually related pictures. Most notably, on the basis of P3 as a similarity indicator, many experiments extended the findings that P3 was observable in semantic priming, which indexed the semantic relatedness between primes and targets. The sensitivity of P3 to semantic priming has been demonstrated not only in mono-modal studies, such as verbal modality (Hill et al., 2002) and pictorial modality (McPherson and Holcomb, 1999), but also in multi-modal studies using pictures and words as stimuli (Watson et al., 2005). The more semantically related between primes and targets, the larger the P3 amplitude. The present study involves the matching between PMs and verbalization forms. When the verbalization form can convey the message of the picture, the similar conceptual representation would be activated due to the high degree of semantic relatedness. Therefore, we hypothesize that the P3 would be observed reflecting similarity and semantic relatedness.

Based on the above literature review, this study, providing electrophysiological evidence, aims to identify effective verbalization forms in representing PMs by examining how the particular verbalization forms match with different-structured PMs. ERP data are recorded while participants view an advertising picture and a following sentence presented word by word. The task is to decide whether the sentence is appropriate or not to describe the picture. Since the extant ERP studies show that N1, P2, and P3 components index some aspects of semantic processing of both verbal and visual stimuli, we would expect these neural components to be observed in the verbo-pictorial matching process for metaphors. And changes of their amplitudes would be indicative of matching extent between verbalization forms and PMs. We hypothesize that enhanced P2, P3 and decreased N1 would be elicited when PMs are matched with effective verbalization forms.

This cognitive study of verbal and pictorial stimuli in advertising is of great significance, because the typical observation time for advertising is 2 s in an editorial context (Pieters and Wedel, 2004). Instead of depending on the conventional self-report or surveys on ad attitudes, establishing the neural changes with a brief exposure to advertising visual stimuli can contribute to the development of both the cognitive study and advertising research of multi-modal metaphors.

## 2. Materials and methods

### 2.1. Participants

Thirty-seven students from Dalian University of Technology participated in the ERP Experiment (24 men, 13 women; average age: 24.2; range: 20–30). All participants were native Chinese speakers with normal or corrected-to-normal vision. They were all right-handed and had no color blindness or color weakness, no history of mental illness or other brain diseases. Informed about the design of the study, all participants gave their written consent for participation before the experiment and received remuneration after the experiment. One participant’s data was excluded for excessive recording artifacts. The experiment was examined and approved by the Biological and Medical Ethics Committee of the Dalian University of Technology.

### 2.2. Stimuli

The stimulus pool included 90 pictures and 180 corresponding Chinese sentences (Table 1 shows representative examples). The pictures fell into three conditions: fusion structure (hereafter as FS), juxtaposition structure (hereafter as JS), and literal structure (hereafter as LS), with 30 pictures in each condition. The source domain and the target domain of each set of three visual structures were the same. The present experiment did not take replacement-structured PM into account considering its complexity in the visual context, the excessive processing load and its scarcity in practical advertising. Instead, literal images that show the product directly (i.e., just images of the advertised objects) are considered, as non-metaphor pictures combined with metaphor headlines are often seen in advertising. The non-metaphor pictures can also be used as a baseline for the condition comparison.

**Table 1:** Examples of stimuli used in the experiment.

Visual structures	Metaphor pictures	Sentences
FS	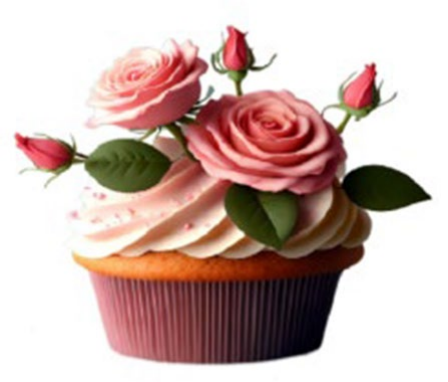	蛋糕是玫瑰。 (Cake is rose.)
		蛋糕像玫瑰。 (Cake is like rose.)
JS	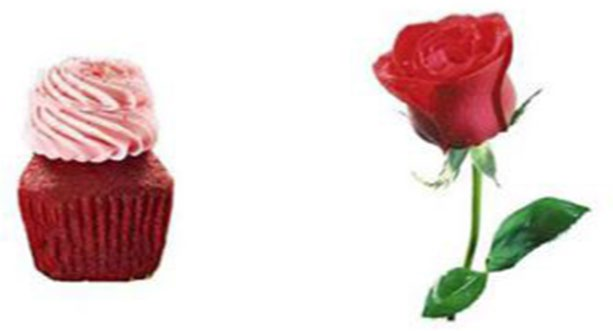	蛋糕是玫瑰。 (Cake is rose.)
		蛋糕像玫瑰。 (Cake is like rose.)
LS	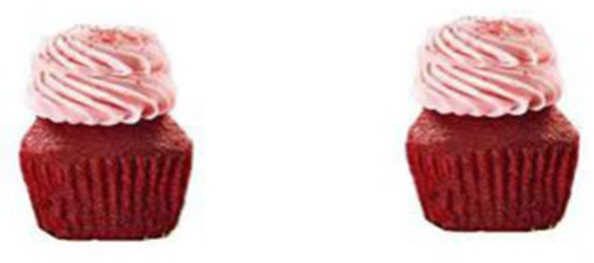	蛋糕是玫瑰。 (Cake is rose.)
		蛋糕像玫瑰。 (Cake is like rose.)

Since the participants in the experiment were all college students, the advertising pictures included the best-selling products of low-involvement, that was, the advertised products in the pictures were what college students could afford and often purchased, including food and electronic devices. In these advertising PMs, trademarks, slogans, and brand names were removed to avoid the impact of reading. As for verbalization form, there were two: “A是(is) B” and “A像(is like) B.” The stimuli were selected based on four pretests.

Initially, 100 fusion-structured adverting PMs were pretested for the selection of appropriate experiment pictures. As familiarity has a modulating influence on the metaphor processing (Blasko and Connine, 1993; Giora and Fein, 1999; Giora, 2003; Blasko and Kazmerski, 2006; Schmidt and Seger, 2009; Mcquire et al., 2017), 70 university students at Dalian University of Technology, China, who did not participate in the later ERP experiment, were invited to rate their familiarity with PM, on a five-point Likert scale ranging from 1 (the least familiar) to 5 (the most familiar) in a preliminary test. Twenty-four highly familiar pictures were removed.

In the second pretest, a naming test (Forceville, 2002) was employed for the identification of the source and target items in PMs. A new group of 10 undergraduates participated in the naming, on a scale from 1 (the least consistent) to 5 (the most consistent). Sixteen pictures of low consistency were deleted.

In addition, a questionnaire was designed and delivered to 45 students to confirm which was the theme (target domain) of the pictures (Forceville, 2002) for the metaphorical expression: A (target domain) 是/像(is/is like) B (source domain). A sentence was presented below each picture, indicating “This is an advertisement for XXX.” The answers were presented in a five-point Likert scale ranging from 1 (do not agree at all) to 5 (agree completely). To avoid redundant cognitive processing, the source and target items in the sentences were named in the same lexical length of Chinese characters.

The fourth is a sentence comprehension test (Tang et al., 2017) conducted to test whether or not the metaphorical sentences could be comprehended, on a five-point Likert scale from 1 (strongly non-comprehensible) to 5 (strongly comprehensible). This questionnaire was delivered to another group of 32 students. Sentences of low comprehensibility were deleted.

Finally, 41 pictures were retained and processed by Adobe Photoshop 13.0 to create the corresponding JS and LS with the same source and target domain. Thirty were used in the formal experiment and another 11 were used in the practice trials. All the pictures had the same luminance, shade and size and the Chinese characters were Song typeface with the same font size.

### 2.3. Procedure

A 3 (pictorial structure: FS, JS, LS) × 2 [verbalization form: A是(is) B, A像(is like) B] within-group experiment was conducted. In total, there were 180 trials, divided into 3 blocks with 60 trials in each. All the stimuli were presented in the center of the screen with white background in a quasi-random order.

**Figure 1:**
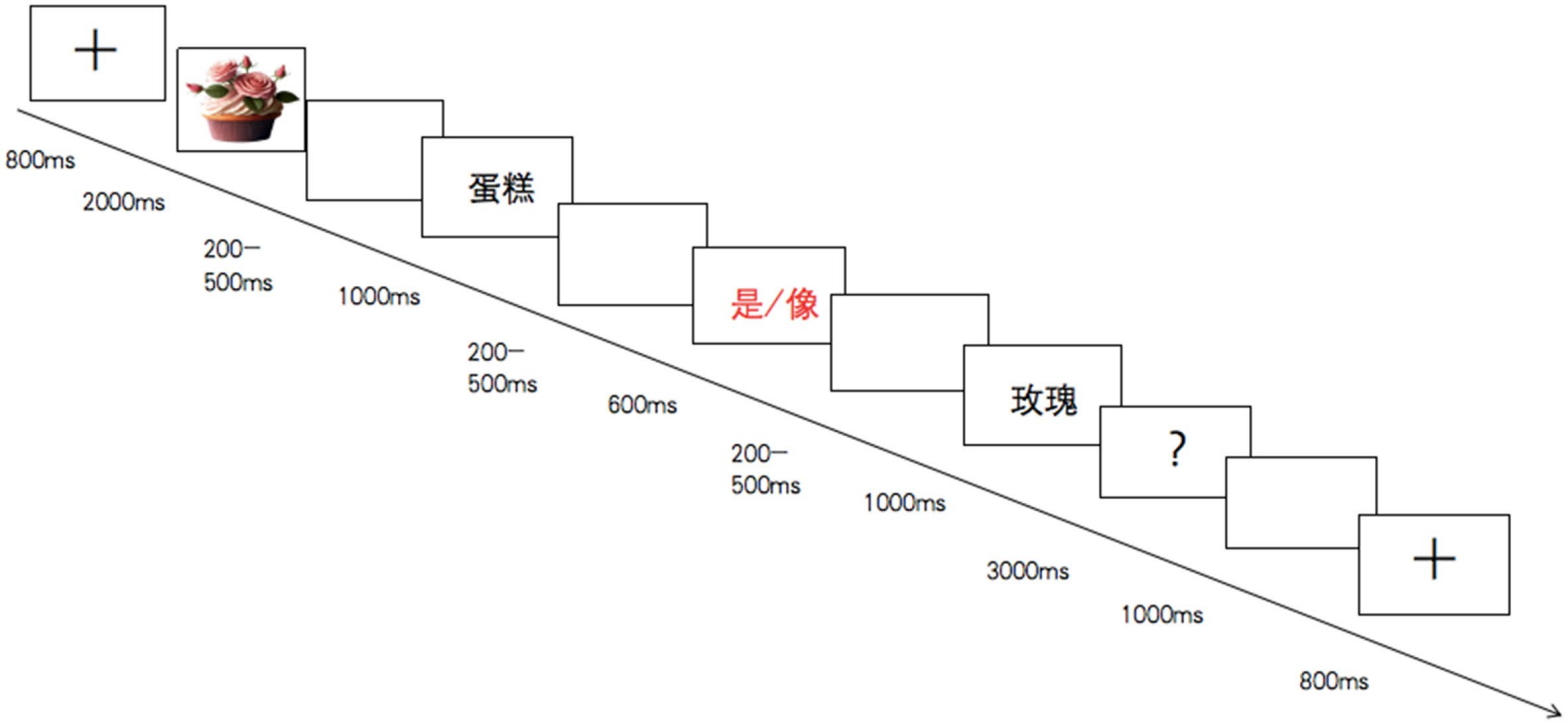
Experimental procedure.

As illustrated in Figure 1, the picture was presented first and then the sentence was presented word by word. The stimuli on each trial were presented in the following time sequence: fixation cross (800 ms), metaphor picture (2,000 ms), blank (200–500 ms), subject (source domain; 1,000 ms), blank (200–500 ms), linking verb [是/像(is/is like) 600 ms], blank (200–500 ms), predicative (target domain, 1,000 ms), and question mark (3,000 ms). At the sight of the question mark, participants needed to make their judgments about whether the sentence was appropriate to describe the picture, by pressing a corresponding key. For counterbalance, half of participants pressed F (appropriate) or J (inappropriate) and the other half of participants pressed J (appropriate) or F (inappropriate). Response period was limited to 3,000 ms and was followed by a 1,000 ms interval. The participants conducted a brief exercise before the experiment.

### 2.4. Electroencephalogram data recording and processing

The participants were seated at a distance of 0.8 m from the screen in a silent room with soft lighting. The tasks were run and data were collected using E-Prime 2.0 (Psychology Software Tools, Sharpsburg, MD). ERPs were time-locked to the onset of the verb [是/像(is/is like)] of the sentence and were obtained by stimulus-locked averaging of the EEG recorded in each condition. Electroencephalogram data were continuously recorded via an electrode cap with 64 Ag/AgCl electrodes, using the international 10/20 system. A 64-channel BrainAmp EEG amplifier (Brain Products, Germany) and the recording software (BrainVision Video Recorder) were used. Electrode impedance was maintained below 10 kΩ in the experiment. EEG was analyzed with Python using the MNE-Python toolbox. The EEG was digitally filtered using FIR filter-based filtering at 0.1–30 Hz band pass. Epochs were 1,000 ms in length with a 200 ms pre-stimulus baseline. ICA was performed after the extraction of the epoch to correct the ocular artifacts. The ground electrode was placed on the forehead and FCz was used as recording reference. Data were re-referenced offline to the average of the two mastoid electrodes.

### 2.5. Statistical analysis

First, the threshold-free cluster-enhancement (TFCE) technique (the exploratory analysis) was used for the analysis of ERP signals to define the region of interest (ROI) and channels by considering all spatial–temporal points. This technique was purely data-driven and took all data of each channel and any time point into account while strictly controlling for multiple comparisons (Mensen and Khatami, 2013). Based on the TFCE results, frontally distributed five electrodes (F3, F1, Fz, F2, and F4) were selected for the analysis of N1 component (100–130 ms); centrally distributed six electrodes (FC3, FCz, FC4, C3, Cz, and C4) for the analysis of P2 component between 180 and 300 ms; six posterior electrodes (P3, Pz, P4, PO3, POz, and PO4) were selected for the analysis of P3 component (330–430 ms). In the next place, we conducted confirmatory analysis (lmer function, package lme4) including the factors pictorial structure and verbalization form as fixed-terms. Significance *p*-values and Type III F-statistics for main effects and interactions for continuous variables (ERP amplitude) were calculated using Satterthwaite approximations to denominator degrees of freedom as implemented in the lme4 (Bates et al., 2014). Planned comparisons and β estimates were calculated using function emmeans as implemented in the package emmeans.

## 3. Results

### 3.1. Behavioral results

The behavioral data were recorded, including the button-press reaction time and the selection frequency of each verbalization form for PM of the same structure. Noticeably, we only recorded the reaction time of the trials in which the sentence was judged to be appropriate for describing the picture.

**Figure 2:**
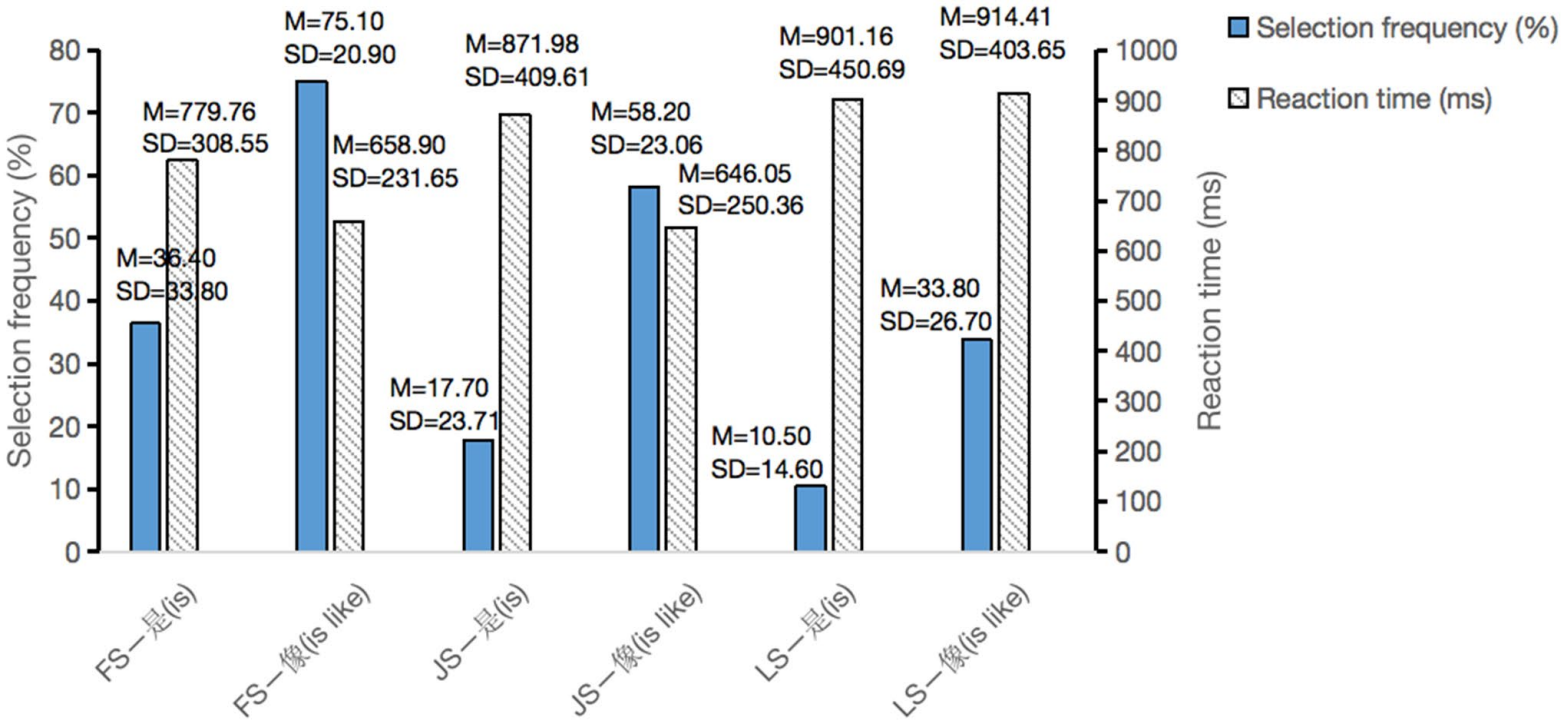
Behavior results.

As can be seen from Figure 2, for three structures of PM, the form “A像(is like) B” is selected more compared to the form “A是(is) B.” And the paired-samples *t*-test further demonstrated that there was a significant difference in selection frequency between two verbalization forms for three structures of advertising pictures (*t* = −6.518, *p* < 0.001 for FS; *t* = −7.976, *p* < 0.001 for JS; *t* = −5.402, *p* < 0.001 for LS). For FS and JS, the reaction time of “A像(is like) B” is shorter than that of “A是(is) B” (t = 2.868, *p* = 0.007 for FS; t = 4.165, *p* < 0.001 for JS). But there was no significant difference between two verbalization forms for LS (*t* = −0.179, *p* = 0.855).

### 3.2. Evoked response potentials results

**Figure 3:**
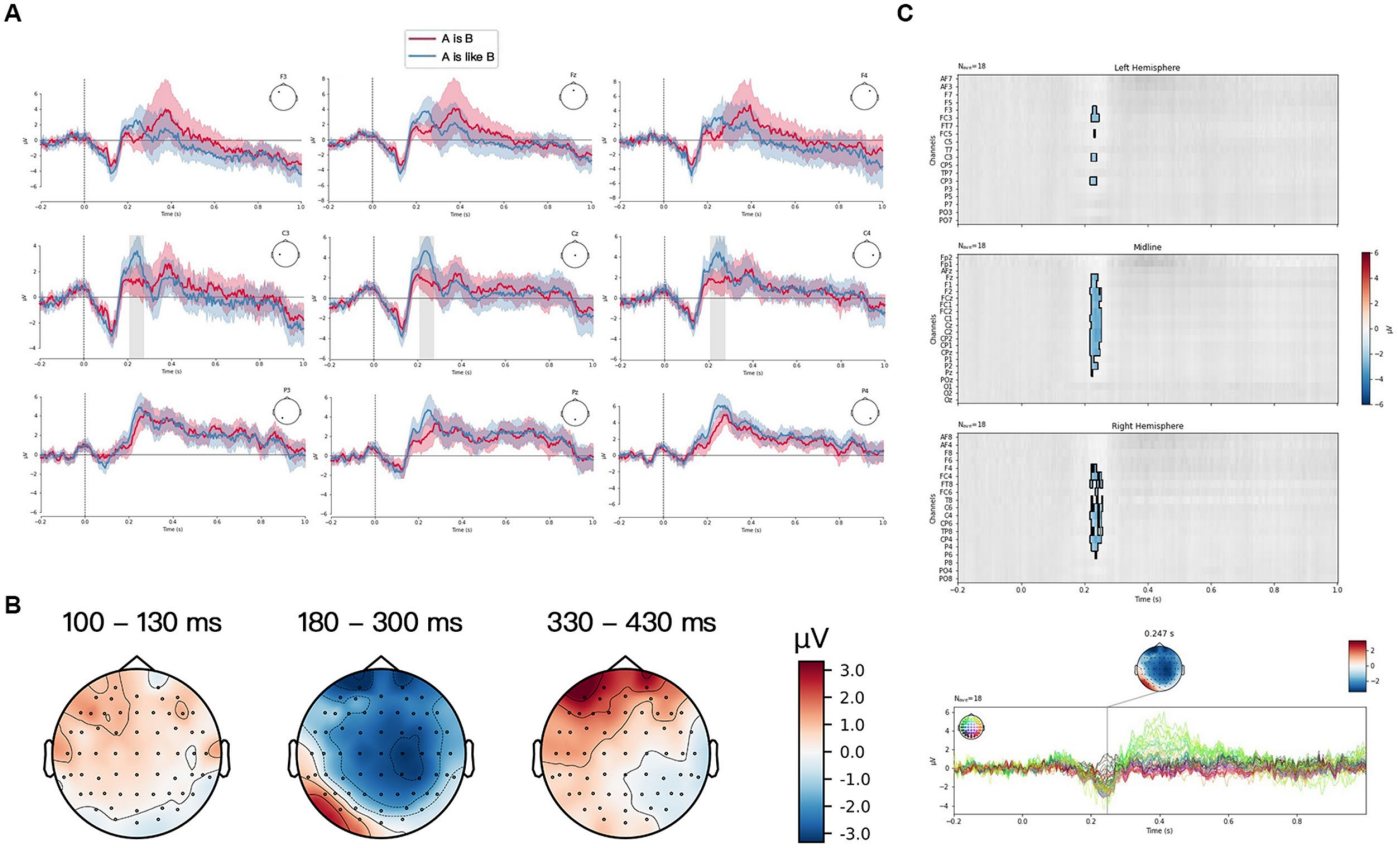
Fusion structure (FS) condition. **(A)** Grand average ERP waveform elicited by “A是(is) B” (red) and “A像(is like) B” (blue) from frontal scalps (F3, Fz, F4), central scalps (C3, Cz, C4) and parietal scalps (P3, Pz, P4). Negative voltage is plotted upwards. **(B)** Scalp distributions of the N1 (100–130 ms), P2 (180–300 ms) and P3 (330–430 ms) based on the difference waves between “A是(is) B” and “A像(is like) B.” **(C)** ERP data analyzed by TFCE.

**Figure 4:**
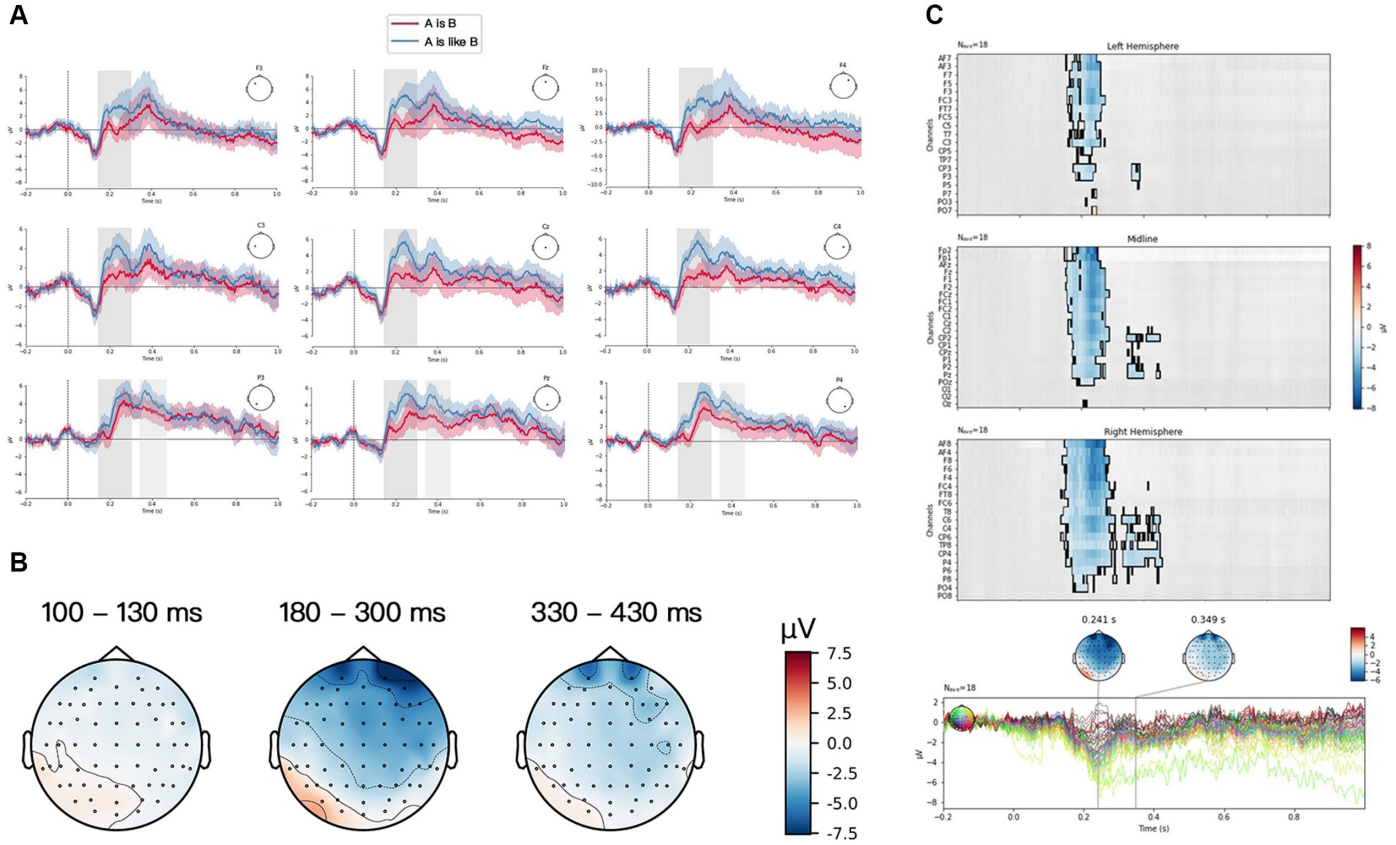
Juxtaposition structure (JS) condition. **(A)** Grand average ERP waveform elicited by “A是(is) B” (red) and “A像(is like) B” (blue) from frontal scalps (F3, Fz, F4), central scalps (C3, Cz, C4) and parietal scalps (P3, Pz, P4). Negative voltage is plotted upwards. **(B)** Scalp distributions of the N1 (100–130 ms), P2 (180–300 ms) and P3 (330–430 ms) based on the difference waves between “A是(is) B” and “A像(is like) B.” **(C)** ERP data analyzed by TFCE.

**Figure 5:**
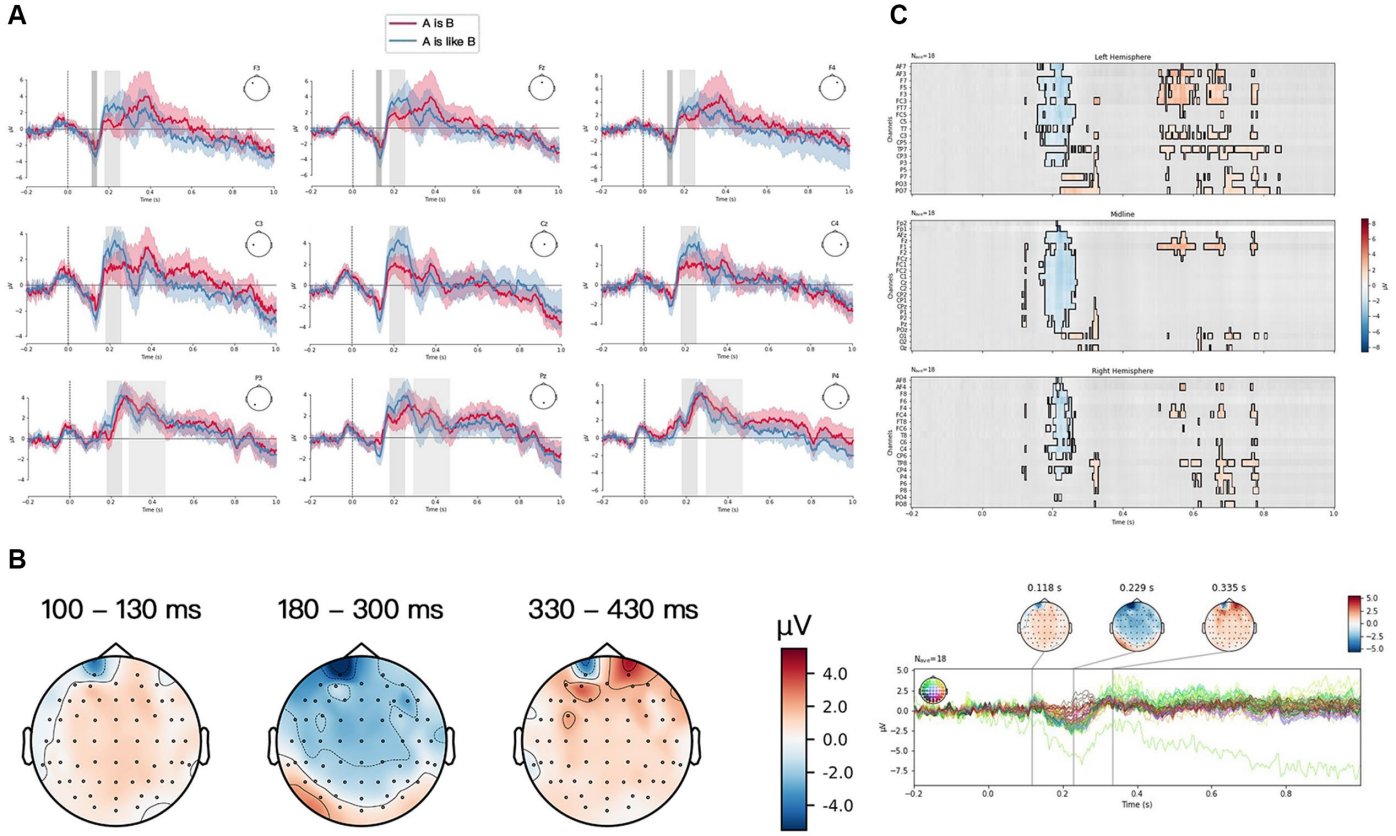
Literal structure (LS) condition. **(A)** Grand average ERP waveform elicited by “A是(is) B” (red) and “A像(is like) B” (blue) from frontal scalps (F3, Fz, F4), central scalps (C3, Cz, C4) and parietal scalps (P3, Pz, P4). Negative voltage is plotted upwards. **(B)** Scalp distributions of the N1 (100–130 ms), P2 (180–300 ms) and P3 (330–430 ms) based on the difference waves between “A是(is) B” and “A像(is like) B.” **(C)** ERP data analyzed by TFCE.

The grand averaged ERP waveform generated by the two verbalization forms [“A是(is) B,” and “A像(is like) B”] for three structures of PM at a frontal group (F3, Fz, F4), a central group (C3, Cz, C4) and a posterior group (P3, Pz, P4) were plotted in Figures 3–5. Visual inspection from TFCE showed the significant difference between two verbalization forms at P2 component for FS in Figure 3. P2 and P3 exhibited significant difference between two forms for JS in Figure 4. And there was significant difference at N1, P2, and P3 components for LS in Figure 5. The topographies of each ERP effect, obtained by subtracting “A像(is like) B” from “A是(is) B” [“A是(is) B”—“A像(is like) B”] from three time frames (100–130 ms, 180–300 ms, 330–430 ms) were also displayed. For FS (see Figure 3), “A像(is like) B” elicited larger P2 than “A是(is) B,” frontally and centrally distributed. For JS (see Figure 4), “A像(is like) B” elicited larger P2 and P3 than “A是(is) B,” frontally distributed. For LS (see Figure 5), “A像(is like) B” elicited larger N1 than “A是(is) B” almost in the whole brain; “A像(is like) B” elicited larger P2 than “A是(is) B,” frontally distributed; “A是(is) B” elicited larger P2 than “A像(is like) B,” focal in the frontal sites.

Table 2 showed the significant difference between the grand averaged ERP waveform generated by the two verbalization forms [“A是(is) B,” and “A像(is like) B”] for three structures of PM. The results of N1 (100–130 ms) indicated the significant main effects of verbalization form [*F* (1, 180) = 4.9547, *p* < 0.05] and pictorial structure [*F* (2, 180) = 7.2407, *p* < 0.001]. The interaction effect between verbalization form and pictorial structure did reach significant [*F* (2, 180) = 7.4031, *p* < 0.001]. N1 generated in the form “A像(is like) B” was significantly larger than that of “A是(is) B” for LS (*β* = −1.735, SD = 0.397, *z* = −4.372, *p* < 0.001), whereas no such difference was observed for FS and JS (*z* = −0.250, *p* > 0.80 for FS; *z* = 0.766, *p* > 0.69 for JS).

**Table 2:** Evoked response potentials (ERP) results.

	N1 (100–130ms)	P2 (180–300 ms)	P3 (330–430 ms)
FS: 是(is) vs像(is like)	*β* = −0.099;	*β* = 1.464;	*β* = 0.218;	SD = 0.397;	SD = 0.376;	SD = 0.484;	*z* = −0.250;	*z* = 3.893;	*z* = 0.451	*p* = 0.803	*p* < 0.001	*p* = 0.652
JS: 是(is) vs像(is like)	*β* = 0.803;	*β* = 2.986;	*β* = 1.166;	SD = 0.397;	SD = 0.376;	SD = 0.484;	*z* = 0.766;	*z* = 7.939;	*z* = 2.410;	*p* = 0.691	*p* < 0.001	*p* = 0.032
LS: 是(is) vs像(is like)	*β* = −1.735;	*β* = 1.248;	*β* = −1.923;	SD = 0.397;	SD = 0.376;	SD = 0.484;	*z* = −4.372;	*z* = 3.317;	*z* = −3.974;	*p* < 0.001	*p* < 0.001	*p* < 0.001

For P2 (180–300 ms) analysis, the results showed that only main effect of verbalization form was significant [*F* (1, 180) = 76.5053, *p* < 0.001]. The effect of pictorial structure was not significant [*F* (2, 180) = 1.7863, *p* > 0.17], whereas the interaction effect between verbalization form and pictorial structure was significant [*F* (2, 180) = 6.3434, *p* = 0.002 < 0.05]. A larger P2 amplitude was observed in the form “A像(is like) B” than that in the form “A是(is) B” (*β* = 1.464, SD = 0.376, *z* = 3.893, *p* < 0.001 for FS; *β* = 2.986, SD = 0.376, *z* = 7.939, *p* < 0.001 for JS; *β* = 1.248, SD = 0.376, *z* = 3.317, *p* < 0.001 for LS).

The results of P3 (330–430 ms) reflected that the interaction effect did reach significant [*F* (2, 180) = 10.694, *p* < 0.001], despite an insignificant main effect of verbalization form [*F* (1, 180) = 0.4131, *p* > 0.52] and pictorial structure [*F* (2, 180) = 2.341, *p* > 0.009]. Follow-up analysis by contrasting two conditions demonstrated that P3 had no significant effect on the two verbalization forms for FS (*z* = 0.451, *p* > 0.65), whereas for JS, “A像(is like) B” elicited significantly larger P3 amplitude than “A是(is) B” (*β* = 1.166, SD = 0.484, *z* = 2.410, *p* = 0.032 < 0.05). And for LS, “A是(is) B” elicited significantly a larger P3 amplitude than “A像(is like) B” (*β* = −1.923, SD = 0.484, *z* = −3.974, *p* < 0.001).

## 4. Discussion

This ERP study investigates the processing of PMs when they are verbalized in different metaphorical expressions, to seek the optimal match between PMs and verbalization forms for the design and application of multi-modal metaphors. A 3 (pictorial structure: FS, JS, LS) × 2 [verbalization form: A是(is) B, A像(is like) B] within-group experiment was conducted among 36 participants to collect behavioral (button-press reaction time and selection frequency), and neural (evoked potentials) data.

Analyzing behavioral performance, the study found that for three structures of advertising PMs, the verbalization form “A像(is like) B” was selected more for illustrating the PMs, compared with the verbalization form “A是(is) B.” On the premise that we recorded the reaction time of the trials in which the sentence was judged to be appropriate for describing the picture, the reaction time of “A是(is) B” for FS and JS was longer. This implies that matching three different-structured advertising PMs with “A是(is) B” was relatively difficult to be comprehended for its recruiting more cognitive effort. In comparison, “A像(is like) B” was easier and more expressive as a verbalization form representing FS and JS PMs. However, there was no significant difference between two verbalization forms for LS. It may reflect certain degree of processing difficulty when LS were verbalized with these two syntactic expressions (which would be discussed in detail later with ERP results).

As for ERP components, the early component N1 and its amplitude change were found in this study. The matching of two verbalization forms with each type of PMs all induced N1 effect, indicating that this initial response observed in the early window was caused by visual stimuli of the experimental pictures with different visual structures. This finding was in line with the previous one (Cao et al., 2018) that both metaphor and literal pictures could elicit N1 due to the perceptual stimulation of the shape of experimental pictures. But for LS, there was significant difference between two verbalization forms. The form “A像(is like) B,” in the judgment task of verbo-pictorial match, evoked a larger amplitude of N1 than “A是(is) B,” indicating that more recognition of incongruity occurred in the processing of “A像(is like) B.” Interestingly, for FS and JS, significant difference was not revealed between two verbalization forms. It may be attributed to the effect of different visual structures of PMs. In the condition of LS, participants could quickly identify the pictured product (target domain) before making the verbo-pictorial matching. While in the condition of FS and JS, the presence of both source domain and target domain made it hard for participants to distinguish them in the short-initial time window, only eliciting the perceptual activation of the picture instead.

Among three structures of PMs, a larger amplitude of P2 was found in “A像(is like) B” than in “A是(is) B.” Previous psycholinguistic studies (Duffy et al., 1989; Hess et al., 1995) found words that were predictable in a sentence context (contextually congruent and semantically associated words) provided greater facilitation than the words that occurred out of context or in incongruent contexts. Electrophysiological results (Biederman and Cooper, 1991) supported these findings, reflected by an enhanced P2 in contextually congruent and semantically associated matching conditions. The similar findings were also found by Federmeier and Kutas (2002), who pointed out that the upcoming pictures could be facilitated by a preceding semantically-related sentences. The more consistent and expected the semantic information, the stronger the P2 effect. In the present experiment, when participants saw the picture and the following sentence “A像(is like) B,” more semantically-related and congruent features were extracted to construct a conceptual mapping between the visual and verbal stimuli. As such, “A像(is like) B,” due to its larger semantic congruity with the PMs, was assumed to be a more effective verbalization form in representing PMs in general.

However, a question remains unclear, i.e., why was “A像(is like) B” the expected syntactic pattern over “A是(is) B”? We believe it is related to their different cognitive semantic features. The syntactic form of “A是(is) B” by nature is one manifestation of Noun Metaphor (Shu, 2002). This type of metaphor is more flexible to be used and also more difficult to be recognized due to the lack of necessary cognitive context, that is, literally provided semantic content alone is not sufficient for establishing a cross-domain mapping. For example, we do not use the ambiguous metaphorical expressions like “Heart is a strawberry” or “Coke is a rocket” in everyday speech, because they were too semantically impenetrable or too difficult to be interpreted without context. The interaction theory of metaphor (Richards, 1965) revealed that the meaning of metaphor came from the interaction of various features of two conceptual domains, including feature selection, emphasis and suppression. It emphasized the process of creating similarity between two concepts in metaphor comprehension. Su et al. (2016) also claimed that metaphor is a process that creates similarity between source domain and target domain in cases where such similarity does not already exist. Whereas “A像(is like) B,” especially with the auxiliary “像(is like),” exactly primes a processing of comparing the target domain with the source domain. As evidenced by Li (1999) and Zhao (2001), in the process of grammaticalization, “像(is like)” has gradually lost its original meaning and become an auxiliary word, which was used to explicitly signal the similarity relation. Therefore, as the terms of the similitude were explicit, the form “A像(is like)B” was more accessible than “A是(is) B.”

In addition, our results showed that both verbalization forms elicited P3 for three structures of PMs. The previous studies reported that P3 amplitude is evoked in case of perceived similarity and semantic relatedness (Picton, 1992; Ma et al., 2008). The higher similarity and semantic relatedness, the stronger P3. There was a significant difference between two verbalization forms for JS and LS in terms of P3 amplitude. The form “A像(is like) B” induced a larger P3 amplitude than the form “A是(is) B” did for JS, whereas for LS, the form “A是(is) B” exhibited stronger P3 than “A像(is like) B.” But there was no significant difference between two forms for FS PM. These results suggested that the feature matching between JS and the verbalization form “A像(is like) B” was more effective, and the same went for the matching between LS and “A是(is) B.” There were more attribute congruity and semantic relatedness for the matching of JS with the form “A像(is like) B” and for LS with the form “A是(is) B” as well. According to these results, it can be concluded that “A像(is like) B” is more effective in representing JS, and “A是(is) B” is more effective for LS. This finding, to some extent, was consistent with the hypothesis proposed by Teng and Sun (2002) that visual construction of juxtaposition expressed the simile relation (i.e., comparison) because displaying two domains beside each other tipped toward similarity-based simile relation, whereas visual templates of one domain was apt to convey the metaphorical relation (i.e., categorization) which mainly results from the transformational effect by blending two different concepts, which enabled viewers to see a concept in terms of another.

So far, two points still need to be noted. The first one was that why we obtained diverging findings about the effective verbalization form for LS as reflected, respectively, in N1, P2 and P3 time window. In the N1 time window, as literal images lacked one domain for conceptual processing of PM, the comparison-based processing route was interfered with. Therefore, the categorization processing [i.e., A是(is) B] occurred. In the P2 time window when the matching process started and was influenced by the contextual predictability, “A像(is like) B” became the dominant and expected. In the P3 time window, based on the feature matching and the deeper information processing, it was confirmed that the more effective form for LS was “A是(is) B.” The second point was that P3 amplitude did not show significant difference between two verbalization forms for FS. We assume that this homogeneity results from the visual construction of FS. The size, the form and the degree to which the two items were fused were different in the experiment, even though four pretests had been carried out. Accordingly, the fusion complexity would have an effect on viewers’ interpretation of PMs, which is likely to hinder the upcoming matching process. This factor of visual complexity should be further controlled in the future studies.

To sum up, the major finding of the present study is that, regardless of the visual structures, the processing of verbalized PM mainly involves the comparison mechanism. Specifically, for both FS and JS, the congruent verbalization form is consistently “A像(is like) B.” Such a major finding can be explained by the career of metaphor theory (Bowdle and Gentner, 2005). This theory postulates that conventional metaphors lead to categorization processes, whereas novel metaphors tend to be processed in the comparison mechanism. And these two processing mechanisms can be represented by two grammatical concordances, “A是(is) B” (categorization) versus “A像(is like) B” (comparison). The advertising metaphor pictures are generally characterized by creativity and low familiarity to viewers. The experimental stimuli used in the current study were PMs of low familiarity. These novel verbo-pictorial information in the categorization mode (is) was highly difficult to comprehend, compared with conventional metaphors that participants are familiar with. Only with a similarity network between the source and the target domain, the comprehension of novel metaphors can be facilitated, that is, when novel metaphors appeared, the comparison processing was generated to access the meaning of novel metaphors because they cannot be categorized based on the prior experience. Accordingly, the comparison processing (is like), i.e., the similarity between the two concrete items in the picture, was more likely to be recognized with fast processing speed with less cognitive load. In contrast, for LS, when matched with verbalization forms, there was a switching of different processing mechanisms reflected by the time course of N1–P2–P3. Only one item (the product) was presented in the preceding LS picture, but two items were referred to in the sentence. At the sight of the sentence, participants, based on their memory retrieval, need to construct an image of the item that is not shown in the picture. In addition, the perception and recognition of PMs would be disturbed, as the mental construction of the absent item varied with each individual. Accordingly, the matching of LS with metaphorical sentences [“A是(is) B” and “A 像(is like) B”] would interfere with the cognitive activities of participants, which was also consistent with the behavioral result that there was no significant difference between two forms for LS. Neither form was effective to verbally represent LS.

## 5. Conclusion

The current research discovered the optimal verbalization form for pictorial metaphors by observing the neuro-cognitive responses in verbo-pictorial matching task. The form “A 像(is like) B” was cognitively preferred across three structures of advertising metaphors. The JS PM verbalized with “A像(is like) B” was the optimal scheme for the design and application of verbo-pictorial metaphor. LS pictures paired with metaphorical sentences would cause the most difficult semantic recognition. Therefore, verbal metaphors are not recommended to be integrated with non-metaphor advertising pictures.

The study not only identifies the early processing mechanism of PMs, but also reveals the verbo-pictorial interaction of metaphor processing. Practically, it provides neural reference for the design of effective multi-modal metaphor in advertising by finding an optimal match between PMs and verbalization forms. In addition to time-domain analysis the study has performed, the future study should employ the time-frequency analysis to study the brain oscillations, such as delta and theta activities, to explore their functional roles for verbo-pictorial metaphor comprehension. A larger sample of the population is also needed, as the participants in this experiment are all university students.

## Data availability statement

The raw data supporting the conclusions of this article will be made available by the authors, without undue reservation.

## Ethics statement

The studies involving human participants were reviewed and approved by Ethics Committee, Dalian University of Technology, China. The patients/participants provided their written informed consent to participate in this study.

## Author contributions

All authors listed have made a substantial, direct, and intellectual contribution to the work and approved it for publication.

## Funding

This work was supported by the National Social Science Foundation of China under Grant Number 19BYY088.

## Conflict of interest

The authors declare that the research was conducted in the absence of any commercial or financial relationships that could be construed as a potential conflict of interest.

## Publisher’s note

All claims expressed in this article are solely those of the authors and do not necessarily represent those of their affiliated organizations, or those of the publisher, the editors and the reviewers. Any product that may be evaluated in this article, or claim that may be made by its manufacturer, is not guaranteed or endorsed by the publisher.

## References

[ref2] Ang S. H. Lim E. A. C. (2006). The influence of metaphors and product type on brand personality perceptions and attitudes. J. Advert. 35, 39–53. doi: 10.1080/00913367.2006.10639226

[ref3] Azizian A. Freitas A. L. Watson T. D. Squires N. K. (2006). Electrophysiological correlates of categorization: P300 amplitude as index of target similarity. Biol. Psychol. 71, 278–288. doi: 10.1016/j.biopsycho.2005.05.002, PMID: 16043279

[ref4] Balconi M. Amenta S. (2010). “A fighter is a lion”. Neuropsychological indexes in comprehending frozen metaphors. J. Pragmat. 42, 3246–3257. doi: 10.1016/j.pragma.2010.06.016

[ref5] Barrett S. E. Rugg M. D. (1990). Event-related potentials and the semantic matching of pictures. Brain Cogn. 14, 201–212. doi: 10.1016/0278-2626(90)90029-N 2285513

[ref6] Bates D. Mächler M. Bolker B. Walker S. (2014). Fitting linear mixed-effects models using lme4. *arXiv* [Epub ahead of preprint].

[ref7] Bergkvist L. Eiderbäck D. Palombo M. (2012). The brand communication effects of using a headline to prompt the key benefit in ads with pictorial metaphors. J. Advert. 41, 67–76. doi: 10.2753/JOA0091-3367410205

[ref8] Biederman I. Cooper E. E. (1991). Object recognition and laterality: null effects. Neuropsychologia 29, 685–694. doi: 10.1016/0028-3932(91)90102-E, PMID: 1944870

[ref9] Blasko D. G. Connine C. M. (1993). Effects of familiarity and aptness on metaphor processing. J. Exp. Psychol. Learn. Mem. Cogn. 19, 295–308. doi: 10.1037/0278-7393.19.2.295, PMID: 7681095

[ref10] Blasko D. G. Kazmerski V. A. (2006). ERP correlates of individual differences in the comprehension of nonliteral language. Metaphor. Symb. 21, 267–284. doi: 10.1207/s15327868ms2104_4

[ref11] Boutros N. N. Korzyukov O. Jansen B. Feingold A. Bell M. (2004). Sensory gating deficits during the mid-latency phase of information processing in medicated schizophrenic patients. Psychiatry Res. 126, 203–215. doi: 10.1016/j.psychres.2004.01.007, PMID: 15157747

[ref12] Bowdle B. F. Gentner D. (2005). The career of metaphor. Psychol. Rev. 112, 193–216. doi: 10.1037/0033-295X.112.1.193 15631593

[ref13] Brennan I. Bahn K. D. (2006). Literal versus extended symbolic messages and advertising effectiveness: the moderating role of need for cognition. Psychol. Mark. 23, 273–295. doi: 10.1002/mar.20111

[ref14] Brookhuis K. A. Mulder G. Mulder L. J. M. Gloerich A. B. M. Van Dellen H. J. Van D. M. J. J.. (1981). Late positive components and stimulus evaluation time. Biol. Psychol. 13, 107–123. doi: 10.1016/0301-0511(81)90030-2 7342984

[ref15] Cao S. Wang Y. Chen H. Wang H. (2018). The N1–N2–LPC pattern in processing advertising pictorial metaphors: an ERP study. Front. Psychol. 9, 1–13. doi: 10.3389/fpsyg.2018.02566, PMID: 30618984 PMC6305598

[ref16] Duffy S. A. Henderson J. M. Morris R. K. (1989). Semantic facilitation of lexical access during sentence processing. J. Exp. Psychol. Learn. Mem. Cognit. 15, 791–801. PMID: 2528603 10.1037//0278-7393.15.5.791

[ref17] Federmeier K. D. Kutas M. (2002). Picture the difference: electrophysiological investigations of picture processing in the two cerebral hemispheres. Neuropsychologia 40, 730–747. doi: 10.1016/S0028-3932(01)00193-2, PMID: 11900725

[ref18] Forceville C. (1996). Pictorial Metaphor in Advertising. London: Routledge.

[ref19] Forceville C. (2002). The identification of target and source in pictorial metaphors. J. Pragmat. 34, 1–14. doi: 10.4324/9780203272305

[ref20] Forceville C. (2008). “Metaphor in pictures and multimodal representations” in The Cambridge Handbook of Metaphor and Thought. ed. Raymond W. G. (Cambridge: Cambridge University Press), 462–482.

[ref21] Forceville C. Urios-Aparisi E. (2009). Multimodal Metaphor (Vol. 11), Berlin, New York: Walter de Gruyter.

[ref22] Gibb H. Wales R. (1990). Metaphor or simile: psychological determinants of the differential use of each sentence form. Metaphor. Symb. 5, 199–213. doi: 10.1207/s15327868ms0504_1

[ref23] Giora R. (2003). On Our Mind. Salience, Context and Figurative Language. New York, NY: Oxford University Press.

[ref24] Giora R. Fein O. (1999). On understanding familiar and less-familiar figurative language. J. Pragmat. 31, 1601–1618. doi: 10.1016/S0378-2166(99)00006-5

[ref25] Gkiouzepas L. Hogg M. K. (2011). Articulating a new framework for visual metaphors in advertising. J. Advert. 40, 103–120. doi: 10.2753/JOA0091-3367400107

[ref26] Gregory M. E. Mergler N. L. (1990). Metaphor comprehension: in search of literal truth, possible sense, and metaphoricity. Metaphor. Symb. 5, 151–173. doi: 10.1207/s15327868ms0503_2

[ref27] Hess D. J. Foss D. J. Carroll P. (1995). Effects of global and local context on lexical processing during language comprehension. J. Exp. Psychol. Gen. 124, 62–82. doi: 10.1037/0096-3445.124.1.62

[ref28] Hill H. Strube M. Roesch-Ely D. Weisbrod M. (2002). Automatic vs. controlled processes in semantic priming-differentiation by event-related potentials. Int. J. Psychophysiol. 44, 197–218. doi: 10.1016/S0167-8760(01)00202-1, PMID: 12031295

[ref200] Indurkhya B. Ojha A. (2013). An empirical study on the role of perceptual similarity in visual metaphors and creativity. Journal of Creative Behavior, 47(3), 233–253. doi: 10.1002/jocb.39

[ref29] Kisley M. A. Noecker T. L. Guinther P. M. (2004). Comparison of sensory gating to mismatch negativity and self-reported perceptual phenomena in healthy adults. Psychophysiology 41, 604–612. doi: 10.1111/j.1469-8986.2004.00191.x, PMID: 15189483

[ref30] Kutas M. Federmeier K. D. (2011). Thirty years and counting: finding meaning in the N400 component of the event related brain potential (ERP). Annu. Rev. Psychol. 62, 621–647. doi: 10.1146/annurev.psych.093008.131123, PMID: 20809790 PMC4052444

[ref31] Kutas M. Hillyard S. A. (1980). Event-related brain potentials to semantically inappropriate and surprisingly large words. Biol. Psychol. 11, 99–116. doi: 10.1016/0301-0511(80)90046-0, PMID: 7272388

[ref32] Lagerwerf L. van Hooijdonk C. M. Korenberg A. (2012). Processing visual rhetoric in advertisements: interpretations determined by verbal anchoring and visual structure. J. Pragmat. 44, 1836–1852. doi: 10.1016/j.pragma.2012.08.009

[ref33] Lakoff G. Johnson M. (2008). Metaphors We Live By, Chicago: University of Chicago Press.

[ref34] Leigh J. H. (1994). The use of figures of speech in print ad headlines. J. Advert. 23, 17–33. doi: 10.1080/00913367.1994.10673439

[ref35] Li X. (1999). “As the same as” and related sentence patterns. Lang. Teach. Res. 3, 85–96.

[ref36] Ma Q. Wang X. Shu L. Dai S. (2008). P300 and categorization in brand extension. Neurosci. Lett. 431, 57–61. doi: 10.1016/j.neulet.2007.11.022, PMID: 18155837

[ref37] Mangun G. R. Hillyard S. A. (1990). Allocation of visual attention to spatial locations: tradeoff functions for event-related brain potentials and detection performance. Percept. Psychophys. 47, 532–550. doi: 10.3758/BF03203106, PMID: 2367174

[ref38] McPherson W. B. Holcomb P. J. (1999). An electrophysiological investigation of semantic priming with pictures of real objects. Psychophysiology 36, 53–65. doi: 10.1017/S0048577299971196, PMID: 10098380

[ref39] McQuarrie E. F. Mick D. G. (1999). Visual rhetoric in advertising: text-interpretive, experimental, and reader-response analyses. J. Consum. Res. 26, 37–54. doi: 10.1086/209549

[ref40] McQuarrie E. F. Phillips B. J. (2005). Indirect persuasion in advertising: how consumers process metaphors presented in pictures and words. J. Advert. 34, 7–20. doi: 10.1080/00913367.2005.10639188

[ref41] Mcquire M. Mccollum L. Chatterjee A. (2017). Aptness and beauty in metaphor. Lang. Cogn. 9, 316–331. doi: 10.1017/langcog.2016.13

[ref42] Mensen A. Khatami R. (2013). Advanced EEG analysis using threshold-free cluster-enhancement and non-parametric statistics. NeuroImage 67, 111–118. doi: 10.1016/j.neuroimage.2012.10.027, PMID: 23123297

[ref250] Mothersbaugh D. L. Huhmann B. A. Franke G.R. (2002). Combinatory and separative effects of rhetorical figures on consumers’ effort and focus in ad processing. Journal of Consumer Research, 28, 589–602. doi: 10.1086/338211

[ref44] Niu Y. N. Wei J. H. Luo Y. J. (2008). Early ERP effects on the scaling of spatial attention in visual search. Prog. Nat. Sci. 18, 381–386. doi: 10.1016/j.pnsc.2007.12.002

[ref45] O’Donnell B. F. Swearer J. M. Smith L. T. Hokama H. McCarley R. W. (1997). A topographic study of ERPs elicited by visual feature discrimination. Brain Topogr. 10, 133–143. doi: 10.1023/A:1022203811678 9455604

[ref46] Ojha A. Indurkhya B. Lee M. (2017). “Is language necessary to interpret visual metaphors?” *in Metaphor in Communication, Science and Education*. eds. Ervas, F., Gola, E. and Rossi, M. (Berlin, Boston: De Gruyter Mouton), 61–76. doi: 10.1515/9783110549928-004

[ref47] Peterson M. O. (2019). Aspects of visual metaphor: an operational typology of visual rhetoric for research in advertising. Int. J. Advert. 38, 67–96. doi: 10.1080/02650487.2018.1447760

[ref48] Phillips B. J. (2000). The impact of verbal anchoring on consumer response to image ads. J. Advert. 29, 15–24. doi: 10.1080/00913367.2000.10673600

[ref49] Phillips B. J. McQuarrie E. F. (2004). Beyond visual metaphor: a new typology of visual rhetoric in advertising. Mark. Theory 4, 113–136. doi: 10.1177/1470593104044089

[ref50] Picton T. W. (1992). The P300 wave of the human event-related potential. J. Clin. Neurophysiol. 9, 456–479. doi: 10.1097/00004691-199210000-00002 1464675

[ref51] Pieters R. Wedel M. (2004). Attention capture and transfer in advertising: brand, pictorial, and text-size effects. J. Mark. 68, 36–50. doi: 10.1509/jmkg.68.2.36.27794

[ref52] Pratarelli M. E. (1994). Semantic processing of pictures and spoken words: evidence from event-related brain potentials. Brain Cogn. 24, 137–157. doi: 10.1006/brcg.1994.1008, PMID: 8123261

[ref350] Pritchard W. S. (1981). Psychophysiology of P300. Psychological Bulletin, 89, 506–540. doi: 10.1037/0033-2909.89.3.506 7255627

[ref300] Pynte J. Besson M. Robichon F.-H. Poli J. (1996). The time-course of metaphor comprehension: An event-related potential study. Memory & Cognition, 24, 356–371. doi: 10.3758/BF03200810 8954602

[ref53] Richards I. A.. The Philosophy of Rhetoric, A Galaxy Book. New York, NY: Oxford University Press, (1965).

[ref54] Rugg M. D. Doyle M. C. (1992). Event-related potentials and recognition memory for low-and high-frequency words. J. Cogn. Neurosci. 4, 69–79. doi: 10.1162/jocn.1992.4.1.69 23967858

[ref55] Ryoo Y. Jeon Y. A. Sung Y. (2021). Interpret me! The interplay between visual metaphors and verbal messages in advertising. Int. J. Advert. 40, 760–782. doi: 10.1080/02650487.2020.1781477

[ref56] Schmidt G. L. Seger C. A. (2009). Neural correlates of metaphor processing: the roles of figurativeness, familiarity and difficulty. Brain Cognit. 71, 375–386. doi: 10.1016/j.bandc.2009.06.001, PMID: 19586700 PMC2783884

[ref57] Song Y. Peng D. L. Xiaolan L. I. Zhang Y. Kang J. Zhe Q. U.. (2007). Neural correlates of short-term perceptual learning in orientation discrimination indexed by event-related potentials. Chin. Sci. Bull. 52, 352–357. doi: 10.1007/s11434-007-0058-7

[ref390] Shu D. (2002). On the operating mechanism of metaphor. Language Science, 4, 11–18.

[ref59] Su C. Tian J. Chen Y. (2016). Latent semantic similarity based interpretation of Chinese metaphors. Eng. Appl. Artif. Intell. 48, 188–203. doi: 10.1016/j.engappai.2015.10.014

[ref60] Tang X. Qi S. Jia X. Wang B. Ren W. (2017). Comprehension of scientific metaphors: complementary processes revealed by ERP. J. Neurolinguistics 42, 12–22. doi: 10.1016/j.jneuroling.2016.11.003

[ref290] Teng N. Y. Sun S. (2002). Grouping, simile, and oxymoron in pictures: A design-based cognitive approach. Metaphor and Symbol, 17, 295–316. doi: 10.1207/S15327868MS1704_3, PMID: 19216017

[ref61] Tlauka M. Clark C. R. Liu P. Conway M. (2009). Encoding modality and spatial memory retrieval. Brain Cogn. 70, 116–122. doi: 10.1016/j.bandc.2009.01.002, PMID: 19216017

[ref62] Van Herten M. Kolk H. H. Chwilla D. J. (2005). An ERP study of P600 effects elicited by semantic anomalies. Cogn. Brain Res. 22, 241–255. doi: 10.1016/j.cogbrainres.2004.09.002 15653297

[ref63] Van Mulken M. Van Hooft A. Nederstigt U. (2014). Finding the tipping point: visual metaphor and conceptual complexity in advertising. J. Advert. 43, 333–343. doi: 10.1080/00913367.2014.920283

[ref64] Vogel E. K. Luck S. J. (2000). The visual N100 component as an index of a discrimination process. Psychophysiology 37, 190–203. doi: 10.1111/1469-8986.3720190, PMID: 10731769

[ref65] Watson T. D. Azizian A. Berry S. Squires N. K. (2005). Event-related potentials as an index of similarity between words and pictures. Psychophysiology 42, 361–368. doi: 10.1111/j.1469-8986.2005.00295.x, PMID: 16008765

[ref66] Zhao J. (2001). On the comparative category of Chinese. J. Chin. Linguist. 5, 1–16.

