# Corrigendum: Matching different-structured advertising pictorial metaphors with verbalization forms: incongruity-based evoked response potentials evidence

**DOI:** 10.3389/fpsyg.2023.1270918

**Published:** 2023-08-11

**Authors:** Shuo Cao, Fang Yue, Shihui Zheng, Yang Fu, Jing Huang, Huili Wang

**Affiliations:** ^1^School of Foreign Languages, Dalian University of Technology, Dalian, China; ^2^Faculty of Management and Economics, Dalian University of Technology, Dalian, China; ^3^Instituto de Neurociencia IUNE, Facultad de Psicología, Universidad de La Laguna, San Cristóbal de La Laguna, Spain; ^4^School of Foreign Languages, Hangzhou City University, Hangzhou, China

**Keywords:** pictorial metaphors, visual structure, verbalization, incongruity, ERP

In the published article, there was an error in affiliation 4. Instead of “School of Foreign Languages, Hangzhou City College, Hangzhou, China”, it should be “School of Foreign Languages, Hangzhou City University, Hangzhou, China”.

The authors apologize for this error and state that this does not change the scientific conclusions of the article in any way. The original article has been updated.

