# Supplementary figures and images for: Matching different-structured advertising pictorial metaphors with verbalization forms: incongruity-based evoked response potentials evidence (part 1 of 2)
Source: Front Psychol. 2023 May 16;14:1131387. doi: 10.3389/fpsyg.2023.1131387 (PMC10227512; doi:10.3389/fpsyg.2023.1131387)

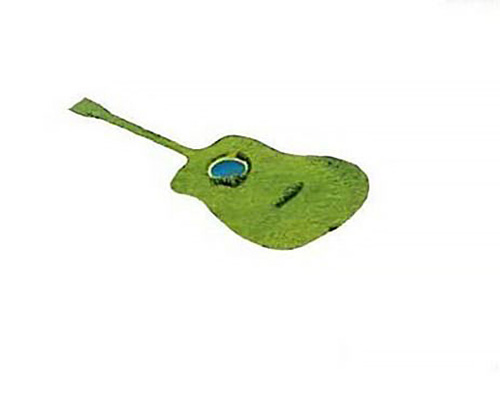

Supplement: Supplementary file 1 [file Presentation_1.zip › Pictures used in the formal experiment/1.1 FS ╝¬╦√ ▓▌╡╪.jpeg]

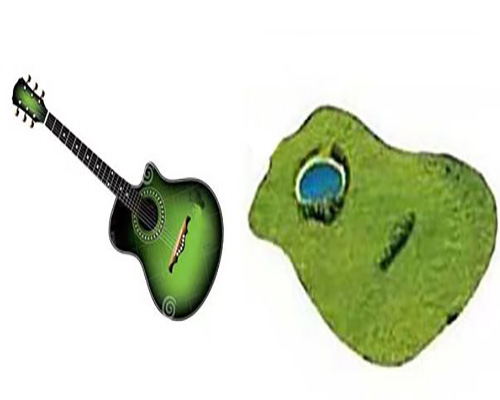

Supplement: Supplementary file 1 [file Presentation_1.zip › Pictures used in the formal experiment/1.2 JS ╝¬╦√ ▓▌╡╪.jpeg]

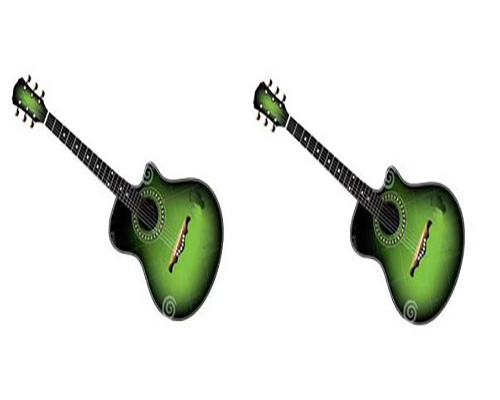

Supplement: Supplementary file 1 [file Presentation_1.zip › Pictures used in the formal experiment/1.3 LS ╝¬╦√ ▓▌╡╪.jpeg]

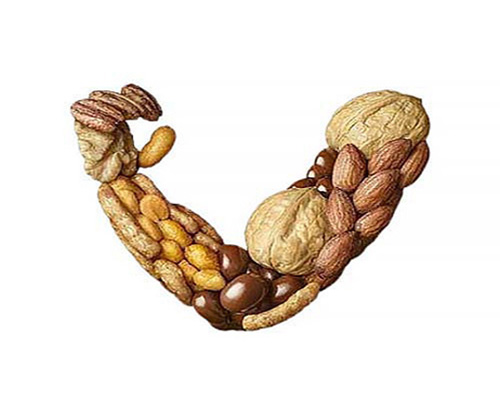

Supplement: Supplementary file 1 [file Presentation_1.zip › Pictures used in the formal experiment/10.1 FS ╝ß╣√ ╝í╚Γ.jpeg]

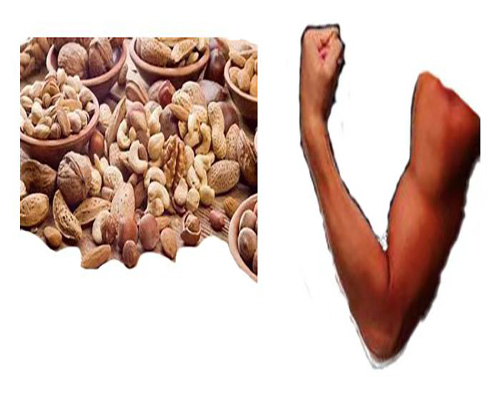

Supplement: Supplementary file 1 [file Presentation_1.zip › Pictures used in the formal experiment/10.2 JS ╝ß╣√ ╝í╚Γ.jpeg]

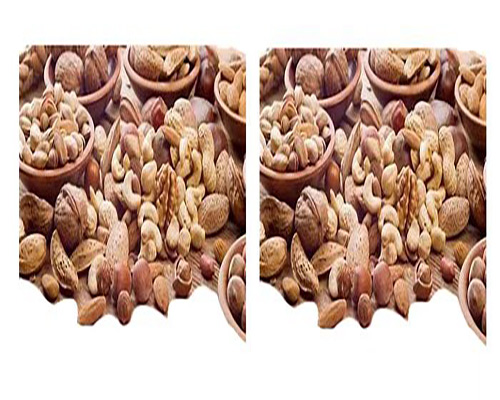

Supplement: Supplementary file 1 [file Presentation_1.zip › Pictures used in the formal experiment/10.3 LS ╝ß╣√ ╝í╚Γ.jpeg]

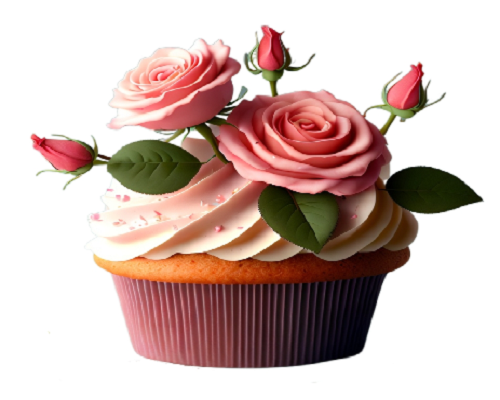

Supplement: Supplementary file 1 [file Presentation_1.zip › Pictures used in the formal experiment/11.1 FS ╡░╕Γ ├╡╣σ.jpeg]

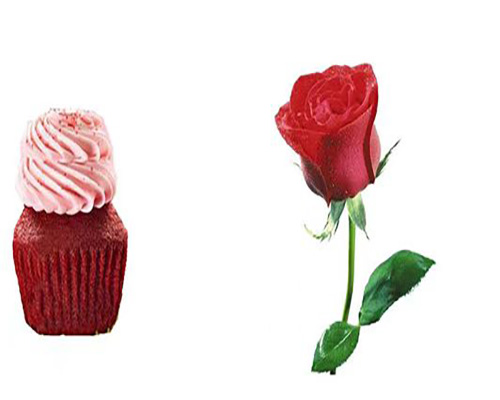

Supplement: Supplementary file 1 [file Presentation_1.zip › Pictures used in the formal experiment/11.2 JS ╡░╕Γ ├╡╣σ.jpeg]

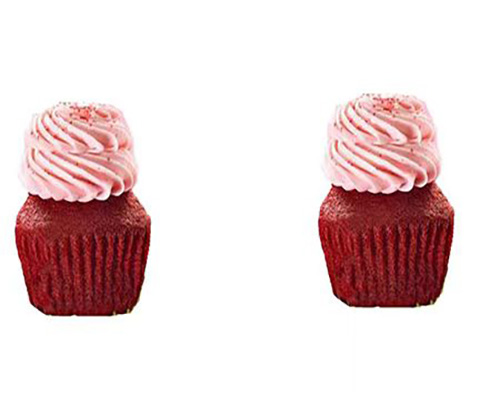

Supplement: Supplementary file 1 [file Presentation_1.zip › Pictures used in the formal experiment/11.3 LS ╡░╕Γ ├╡╣σ.jpeg]

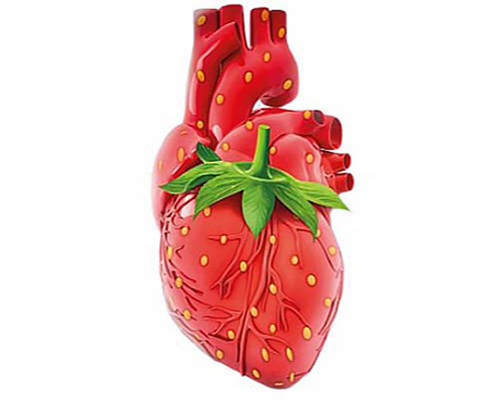

Supplement: Supplementary file 1 [file Presentation_1.zip › Pictures used in the formal experiment/12.1 FS ╨─╘α ▓▌▌«.jpeg]

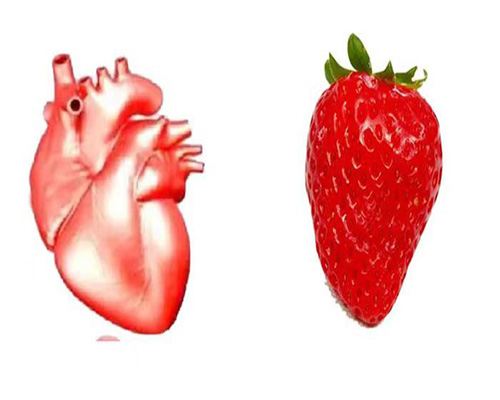

Supplement: Supplementary file 1 [file Presentation_1.zip › Pictures used in the formal experiment/12.2 JS ╨─╘α ▓▌▌«.jpeg]

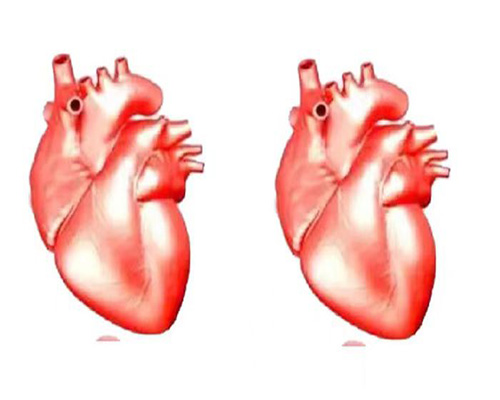

Supplement: Supplementary file 1 [file Presentation_1.zip › Pictures used in the formal experiment/12.3 LS ╨─╘α ▓▌▌«.jpeg]

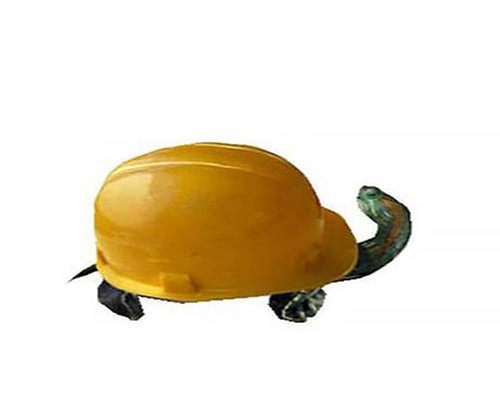

Supplement: Supplementary file 1 [file Presentation_1.zip › Pictures used in the formal experiment/13.1 FS ░▓╚1⁄2├▒ ╬┌╣Ω┐╟.jpeg]

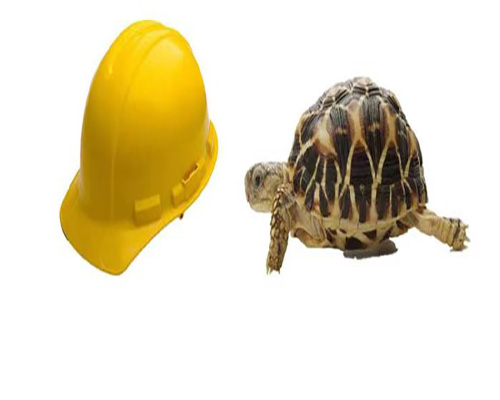

Supplement: Supplementary file 1 [file Presentation_1.zip › Pictures used in the formal experiment/13.2 JS ░▓╚1⁄2├▒ ╬┌╣Ω┐╟.jpeg]

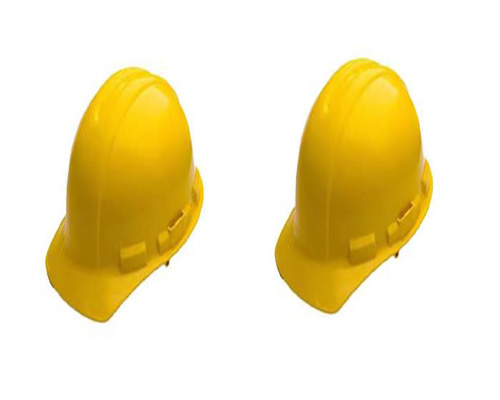

Supplement: Supplementary file 1 [file Presentation_1.zip › Pictures used in the formal experiment/13.3 LS ░▓╚1⁄2├▒ ╬┌╣Ω┐╟.jpeg]

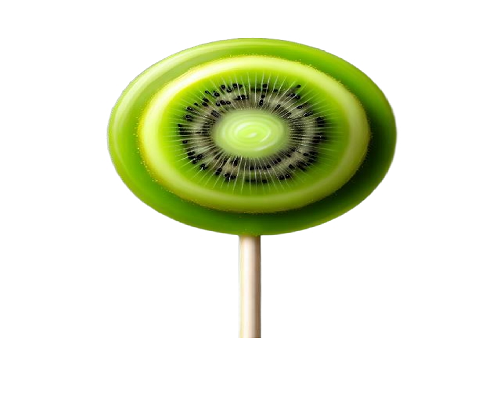

Supplement: Supplementary file 1 [file Presentation_1.zip › Pictures used in the formal experiment/14.1 FS Γ¿║∩╠╥ ░⌠░⌠╠╟.jpeg]

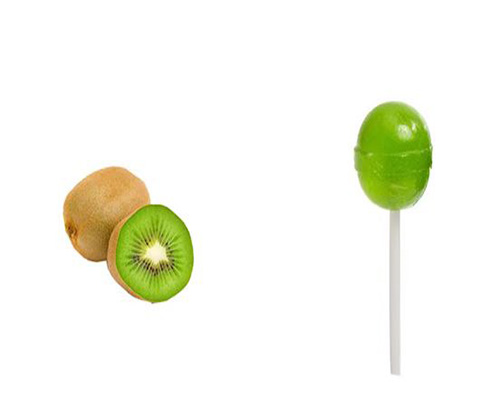

Supplement: Supplementary file 1 [file Presentation_1.zip › Pictures used in the formal experiment/14.2 JS Γ¿║∩╠╥ ░⌠░⌠╠╟.jpeg]

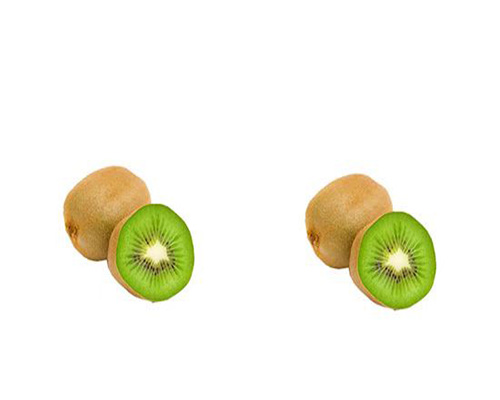

Supplement: Supplementary file 1 [file Presentation_1.zip › Pictures used in the formal experiment/14.3 LS Γ¿║∩╠╥ ░⌠░⌠╠╟.jpeg]

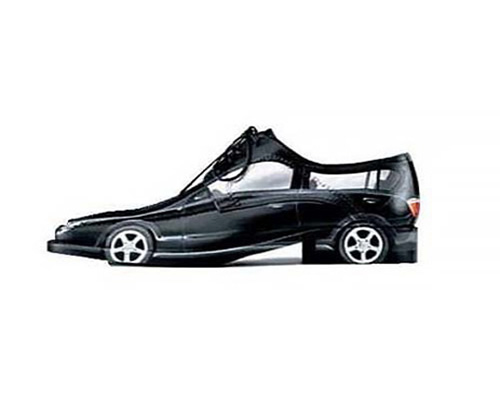

Supplement: Supplementary file 1 [file Presentation_1.zip › Pictures used in the formal experiment/15.1 FS ╜╬│╡ ╞ñ╨1⁄4.jpeg]

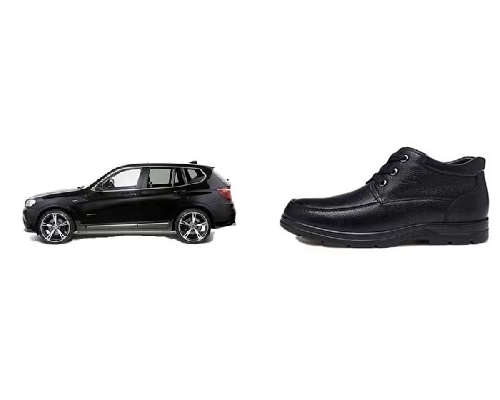

Supplement: Supplementary file 1 [file Presentation_1.zip › Pictures used in the formal experiment/15.2 JS ╜╬│╡ ╞ñ╨1⁄4.jpeg]

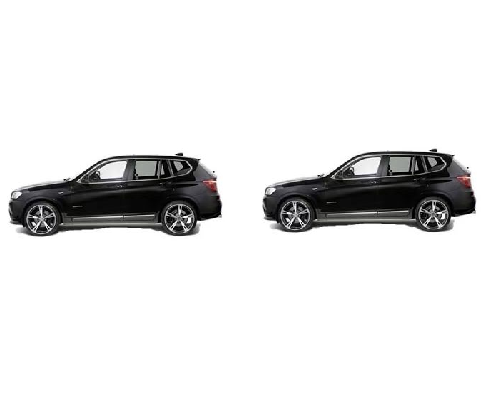

Supplement: Supplementary file 1 [file Presentation_1.zip › Pictures used in the formal experiment/15.3 LS ╜╬│╡ ╞ñ╨1⁄4.jpeg]

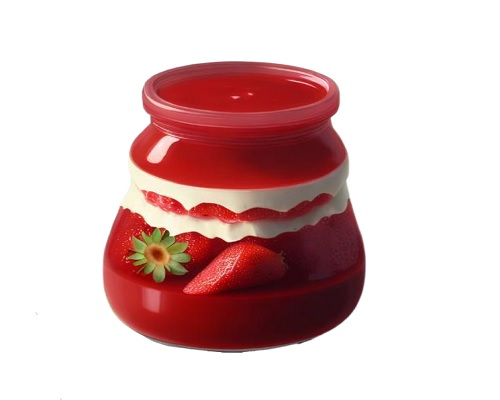

Supplement: Supplementary file 1 [file Presentation_1.zip › Pictures used in the formal experiment/16.1 FS ╣√╜┤ ▓▌▌«.jpeg]

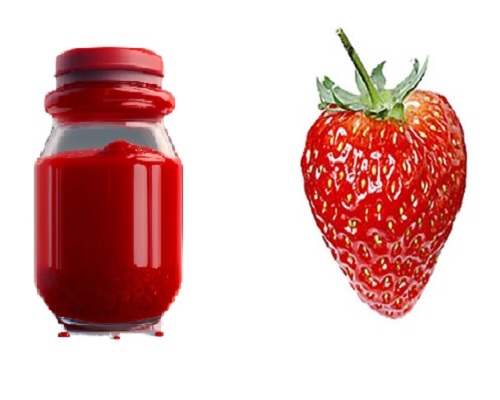

Supplement: Supplementary file 1 [file Presentation_1.zip › Pictures used in the formal experiment/16.2 JS ╣√╜┤ ▓▌▌«.jpeg]

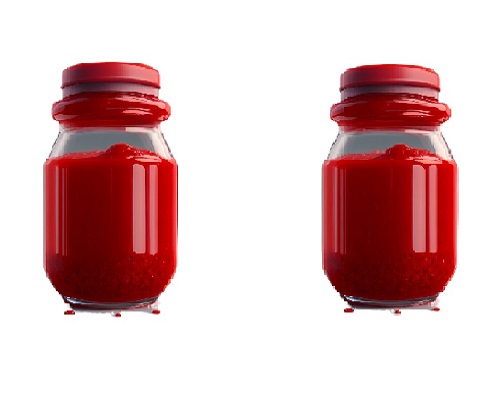

Supplement: Supplementary file 1 [file Presentation_1.zip › Pictures used in the formal experiment/16.3 LS ╣√╜┤ ▓▌▌«.jpeg]

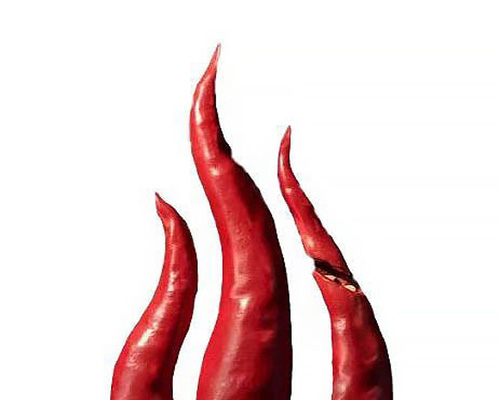

Supplement: Supplementary file 1 [file Presentation_1.zip › Pictures used in the formal experiment/17.1 FS └▒╜╖ ╗≡╤μ.jpeg]

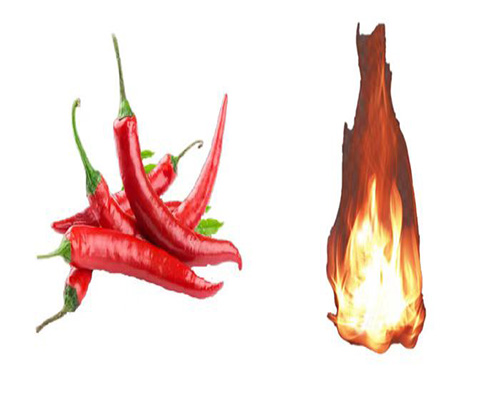

Supplement: Supplementary file 1 [file Presentation_1.zip › Pictures used in the formal experiment/17.2 JS └▒╜╖ ╗≡╤μ.jpeg]

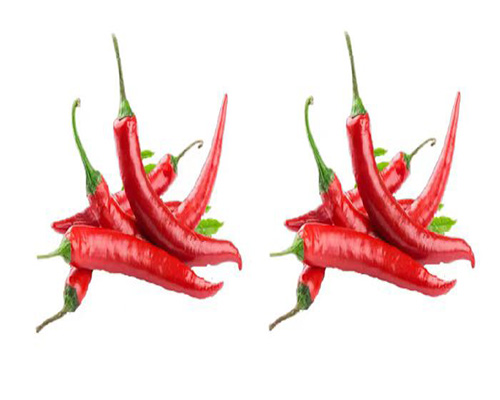

Supplement: Supplementary file 1 [file Presentation_1.zip › Pictures used in the formal experiment/17.3 LS └▒╜╖ ╗≡╤μ.jpeg]

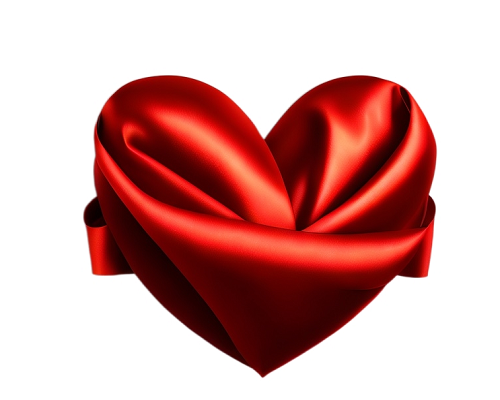

Supplement: Supplementary file 1 [file Presentation_1.zip › Pictures used in the formal experiment/18.1 FS ╨─╘α │±┤°.jpeg]

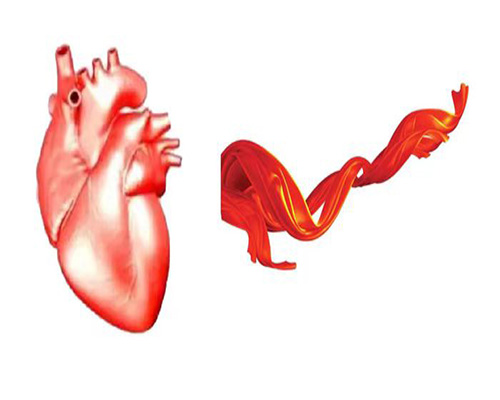

Supplement: Supplementary file 1 [file Presentation_1.zip › Pictures used in the formal experiment/18.2 JS ╨─╘α │±┤°.jpeg]

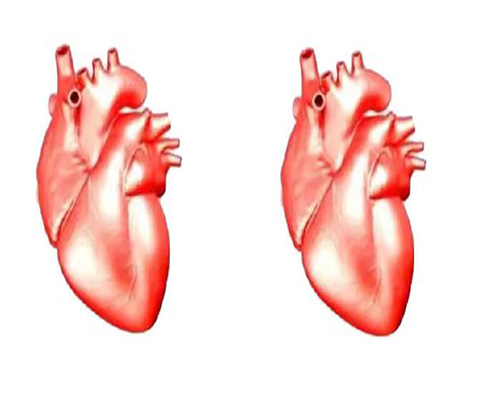

Supplement: Supplementary file 1 [file Presentation_1.zip › Pictures used in the formal experiment/18.3 LS ╨─╘α │±┤°.jpeg]

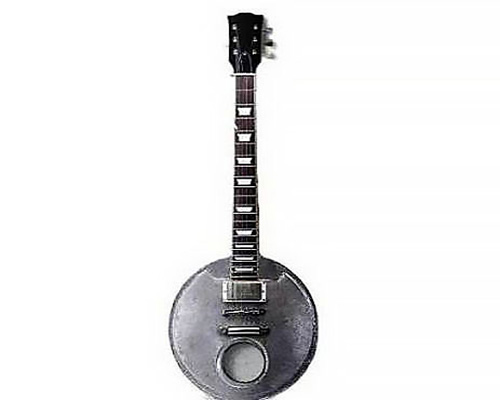

Supplement: Supplementary file 1 [file Presentation_1.zip › Pictures used in the formal experiment/19.1 FS ╝¬╦√ ╘┐│╫.jpeg]

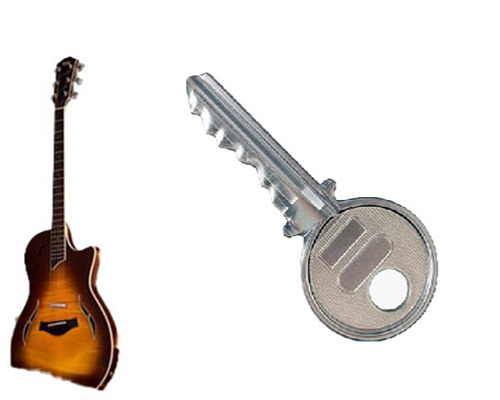

Supplement: Supplementary file 1 [file Presentation_1.zip › Pictures used in the formal experiment/19.2 JS ╝¬╦√ ╘┐│╫.jpeg]

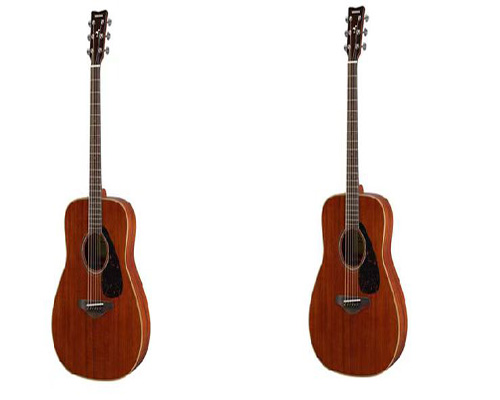

Supplement: Supplementary file 1 [file Presentation_1.zip › Pictures used in the formal experiment/19.3 LS ╝¬╦√ ╘┐│╫.jpeg]

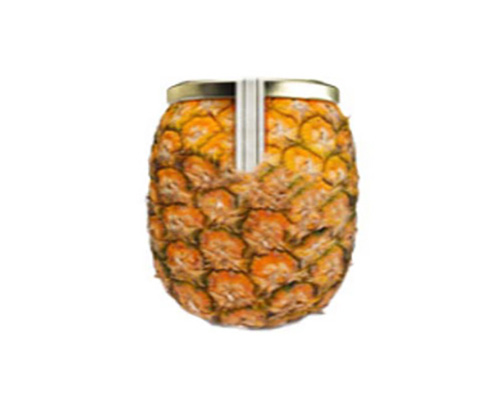

Supplement: Supplementary file 1 [file Presentation_1.zip › Pictures used in the formal experiment/2.1 FS ▓ñ┬▄ ╣▐═╖.jpeg]

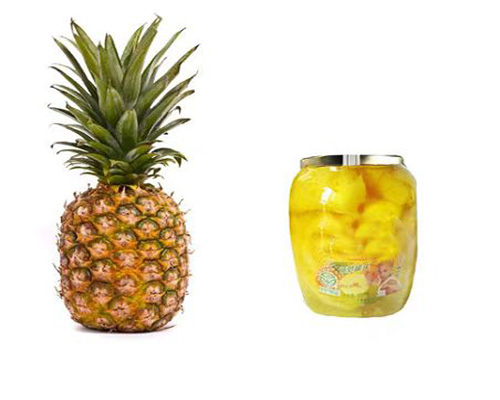

Supplement: Supplementary file 1 [file Presentation_1.zip › Pictures used in the formal experiment/2.2 JS ▓ñ┬▄ ╣▐═╖.jpeg]

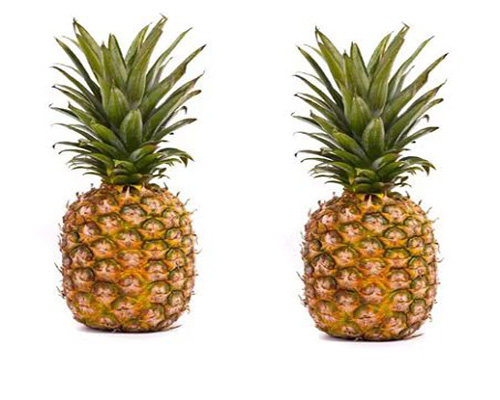

Supplement: Supplementary file 1 [file Presentation_1.zip › Pictures used in the formal experiment/2.3 LS ▓ñ┬▄ ╣▐═╖.jpeg]

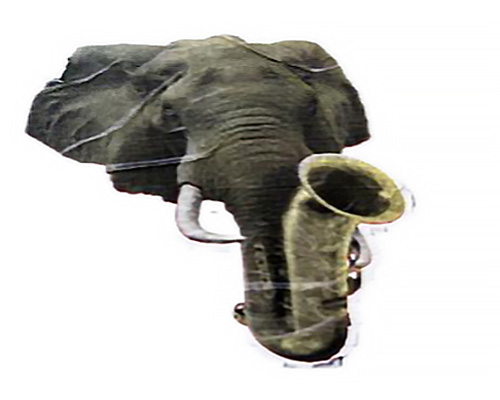

Supplement: Supplementary file 1 [file Presentation_1.zip › Pictures used in the formal experiment/20.1 FS ┤≤╧≤ ║┼╜╟.jpeg]

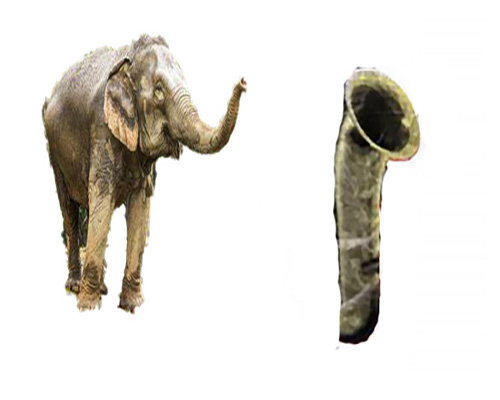

Supplement: Supplementary file 1 [file Presentation_1.zip › Pictures used in the formal experiment/20.2 JS ┤≤╧≤ ║┼╜╟.jpeg]

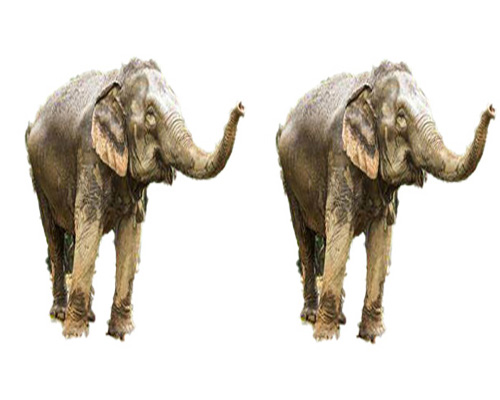

Supplement: Supplementary file 1 [file Presentation_1.zip › Pictures used in the formal experiment/20.3 LS ┤≤╧≤ ║┼╜╟.jpeg]

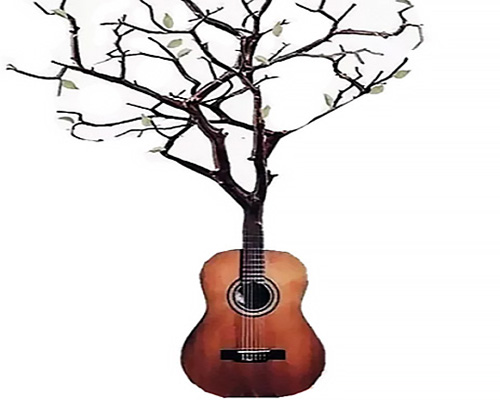

Supplement: Supplementary file 1 [file Presentation_1.zip › Pictures used in the formal experiment/21.1 FS ╝¬╦√ ┤≤╩≈.jpeg]

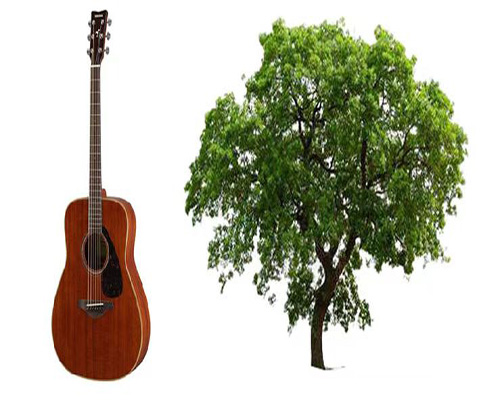

Supplement: Supplementary file 1 [file Presentation_1.zip › Pictures used in the formal experiment/21.2 JS ╝¬╦√ ┤≤╩≈.jpeg]

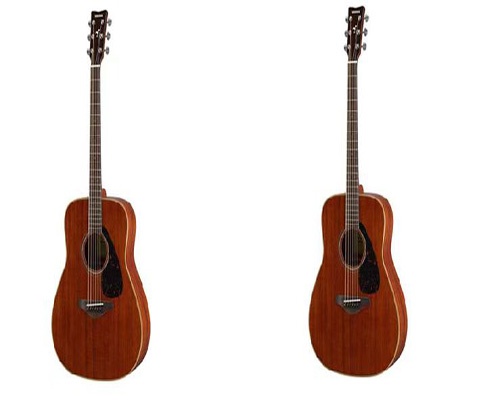

Supplement: Supplementary file 1 [file Presentation_1.zip › Pictures used in the formal experiment/21.3 LS ╝¬╦√ ┤≤╩≈.jpeg]

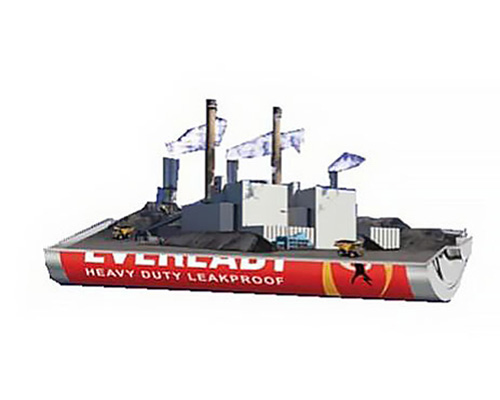

Supplement: Supplementary file 1 [file Presentation_1.zip › Pictures used in the formal experiment/22.1 FS ╣ñ│o ╡τ│╪.jpeg]

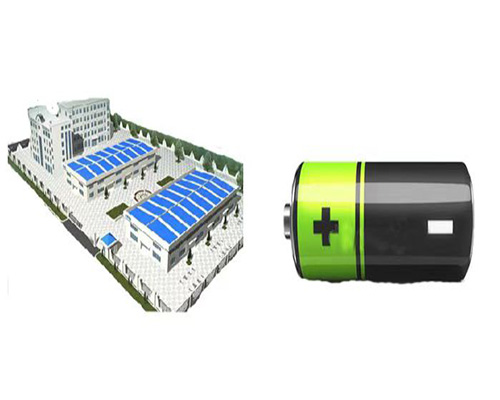

Supplement: Supplementary file 1 [file Presentation_1.zip › Pictures used in the formal experiment/22.2 JS ╣ñ│o ╡τ│╪.jpeg]

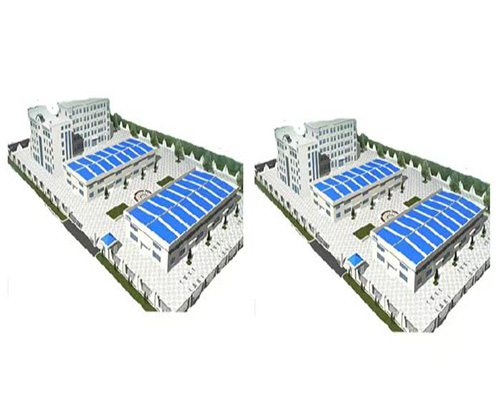

Supplement: Supplementary file 1 [file Presentation_1.zip › Pictures used in the formal experiment/22.3 LS ╣ñ│o ╡τ│╪.jpeg]

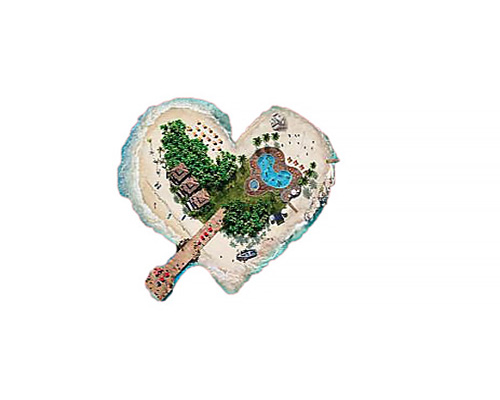

Supplement: Supplementary file 1 [file Presentation_1.zip › Pictures used in the formal experiment/23.1 FS ╡║╙∞ ░«╨─.jpeg]

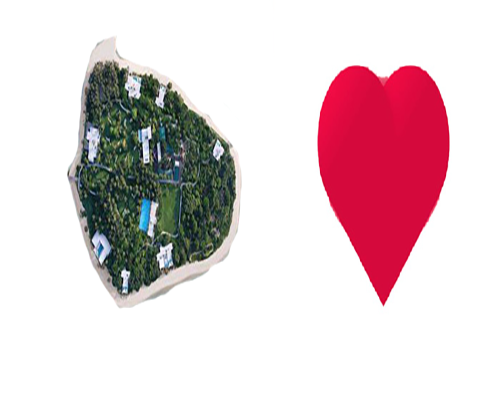

Supplement: Supplementary file 1 [file Presentation_1.zip › Pictures used in the formal experiment/23.2 JS ╡║╙∞ ░«╨─.jpeg]

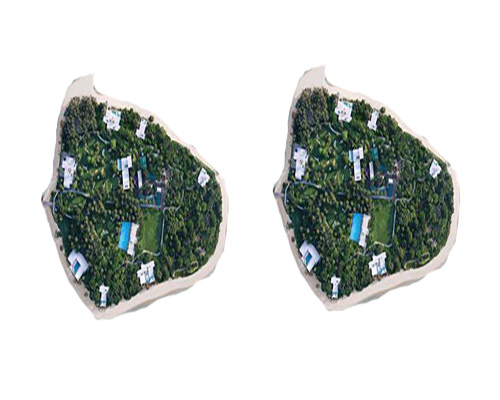

Supplement: Supplementary file 1 [file Presentation_1.zip › Pictures used in the formal experiment/23.3 LS ╡║╙∞ ░«╨─.jpeg]

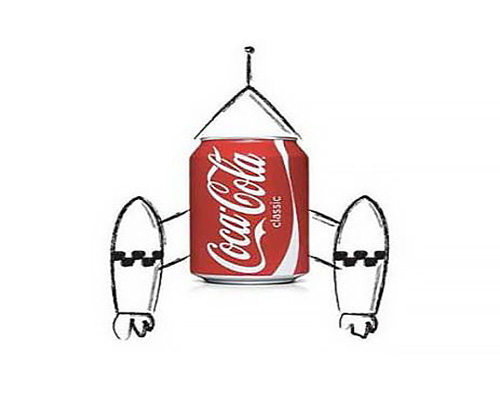

Supplement: Supplementary file 1 [file Presentation_1.zip › Pictures used in the formal experiment/24.1 FS ┐╔└╓ ╗≡╝2.jpeg]

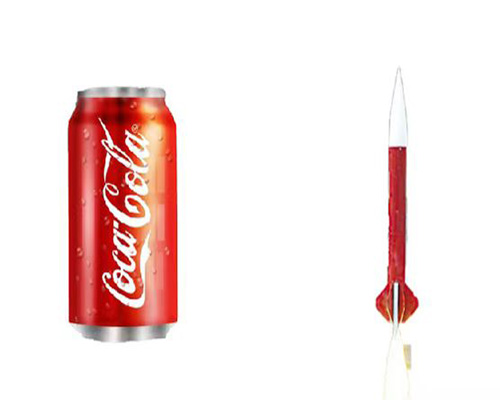

Supplement: Supplementary file 1 [file Presentation_1.zip › Pictures used in the formal experiment/24.2 JS ┐╔└╓ ╗≡╝2.jpeg]

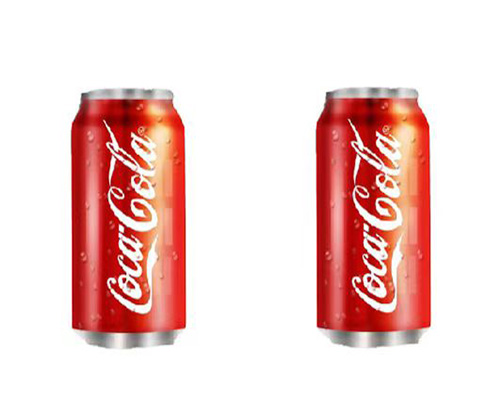

Supplement: Supplementary file 1 [file Presentation_1.zip › Pictures used in the formal experiment/24.3 LS ┐╔└╓ ╗≡╝2.jpeg]

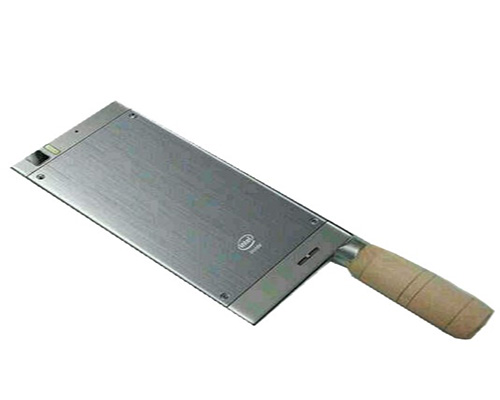

Supplement: Supplementary file 1 [file Presentation_1.zip › Pictures used in the formal experiment/25.1 FS ▓╦╡╢ ╩╓╗·.jpeg]

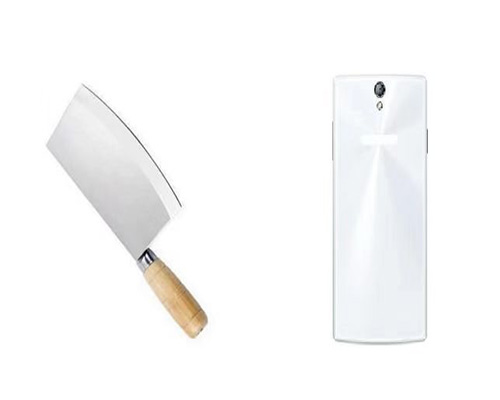

Supplement: Supplementary file 1 [file Presentation_1.zip › Pictures used in the formal experiment/25.2 JS ▓╦╡╢ ╩╓╗·.jpeg]

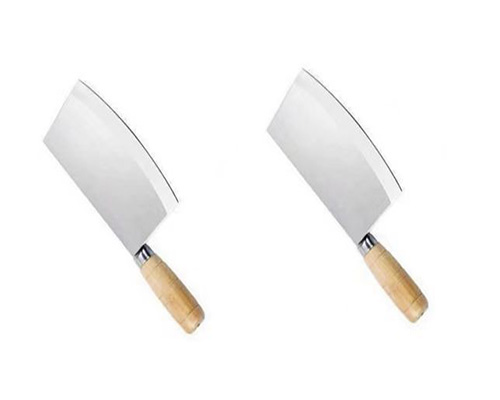

Supplement: Supplementary file 1 [file Presentation_1.zip › Pictures used in the formal experiment/25.3 LS ▓╦╡╢ ╩╓╗·.jpeg]

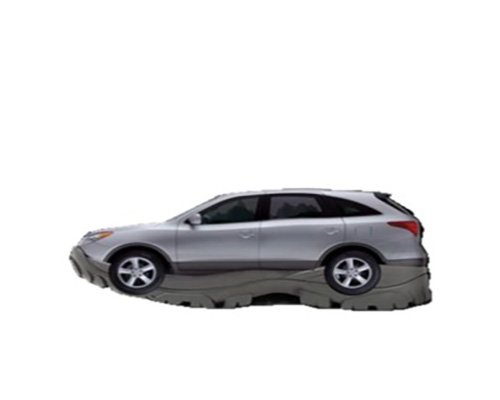

Supplement: Supplementary file 1 [file Presentation_1.zip › Pictures used in the formal experiment/26.1 FS ╘╦╢»╨1⁄4 ╦╜╝╥│╡.jpeg]

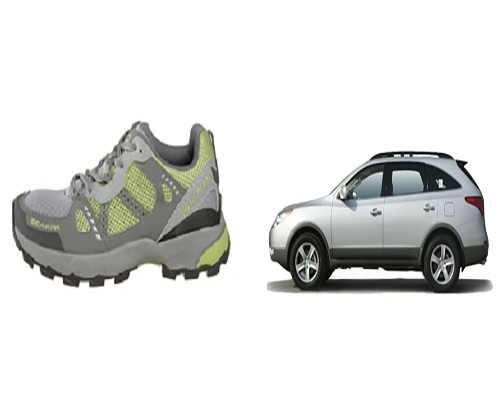

Supplement: Supplementary file 1 [file Presentation_1.zip › Pictures used in the formal experiment/26.2 JS ╘╦╢»╨1⁄4 ╦╜╝╥│╡.jpeg]

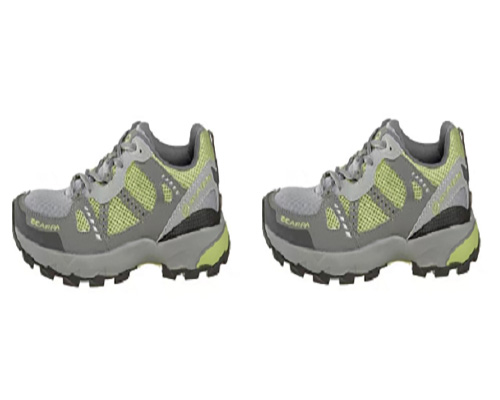

Supplement: Supplementary file 1 [file Presentation_1.zip › Pictures used in the formal experiment/26.3 LS ╘╦╢»╨1⁄4 ╦╜╝╥│╡.jpeg]

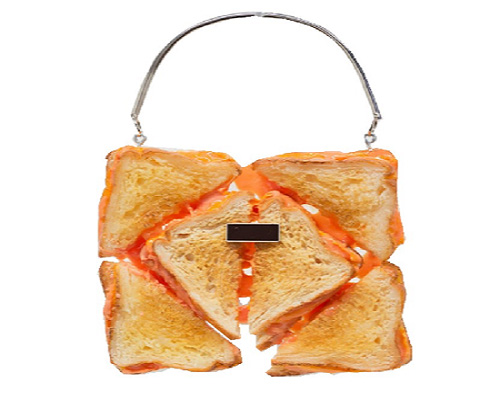

Supplement: Supplementary file 1 [file Presentation_1.zip › Pictures used in the formal experiment/27.1 FS ├μ░n╞1⁄4 ╩╓╠ß░n.jpeg]

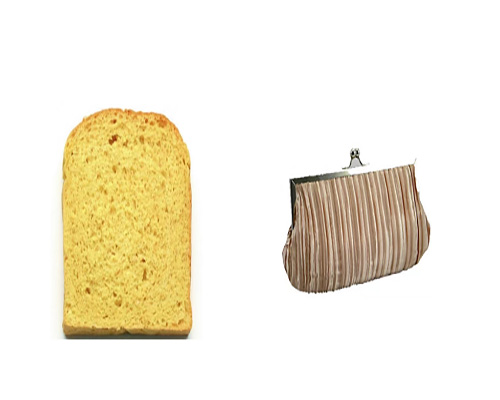

Supplement: Supplementary file 1 [file Presentation_1.zip › Pictures used in the formal experiment/27.2 JS ├μ░n╞1⁄4 ╩╓╠ß░n.jpeg]

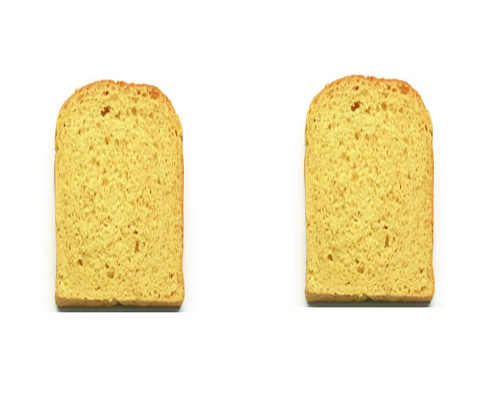

Supplement: Supplementary file 1 [file Presentation_1.zip › Pictures used in the formal experiment/27.3 LS ├μ░n╞1⁄4 ╩╓╠ß░n.jpeg]

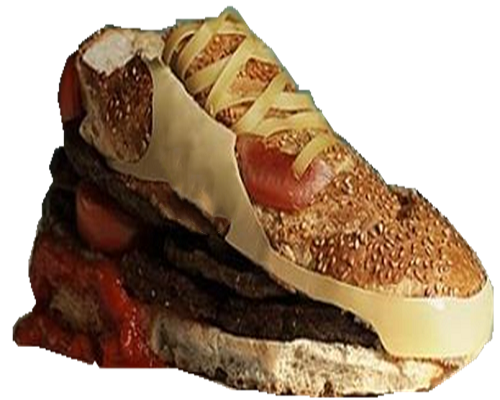

Supplement: Supplementary file 1 [file Presentation_1.zip › Pictures used in the formal experiment/28.1 FS ║║▒ñ ╨1⁄4╫╙.jpeg]

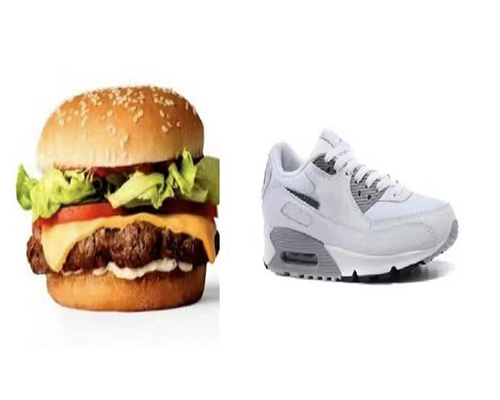

Supplement: Supplementary file 1 [file Presentation_1.zip › Pictures used in the formal experiment/28.2 JS ║║▒ñ ╨1⁄4╫╙.jpeg]

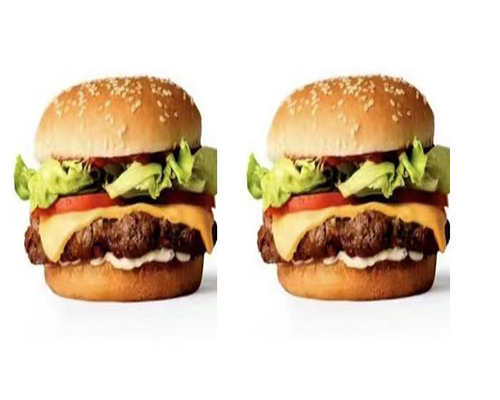

Supplement: Supplementary file 1 [file Presentation_1.zip › Pictures used in the formal experiment/28.3 LS ║║▒ñ ╨1⁄4╫╙.jpeg]

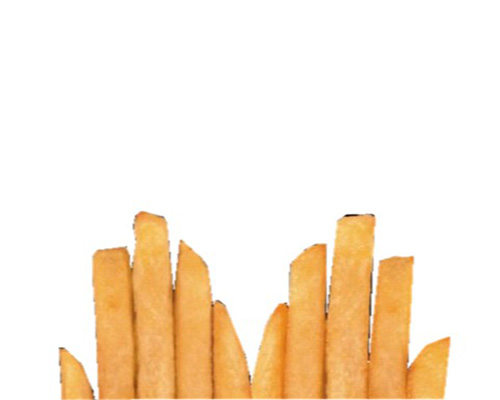

Supplement: Supplementary file 1 [file Presentation_1.zip › Pictures used in the formal experiment/29.1 FS ╩φ╠⌡ ╩╓╓╕.jpeg]

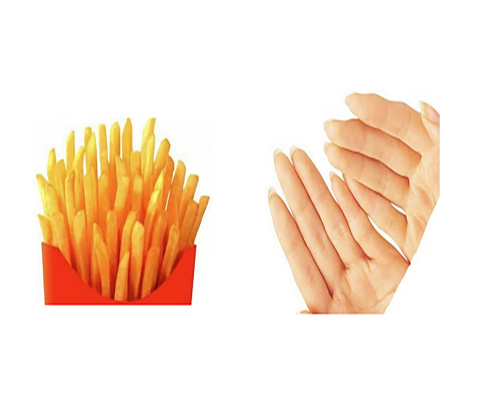

Supplement: Supplementary file 1 [file Presentation_1.zip › Pictures used in the formal experiment/29.2 JS ╩φ╠⌡ ╩╓╓╕.jpeg]

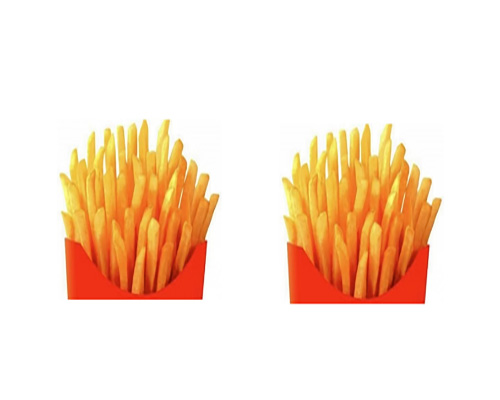

Supplement: Supplementary file 1 [file Presentation_1.zip › Pictures used in the formal experiment/29.3 LS ╩φ╠⌡ ╩╓╓╕.jpeg]

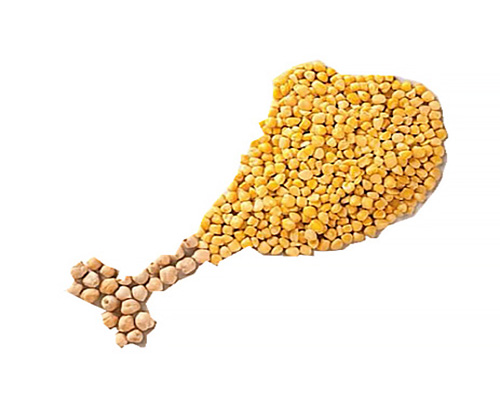

Supplement: Supplementary file 1 [file Presentation_1.zip › Pictures used in the formal experiment/3.1 FS ╘╙┴╕-╝a═╚.jpeg]

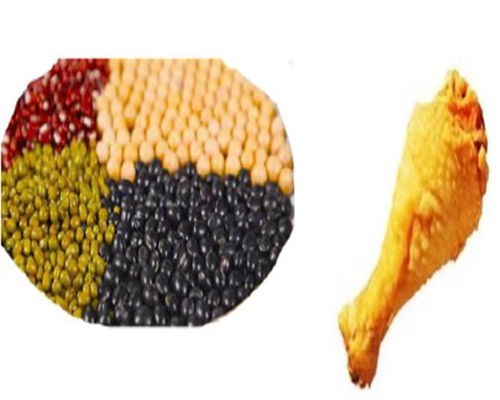

Supplement: Supplementary file 1 [file Presentation_1.zip › Pictures used in the formal experiment/3.2 JS ╘╙┴╕ ╝a═╚.jpeg]

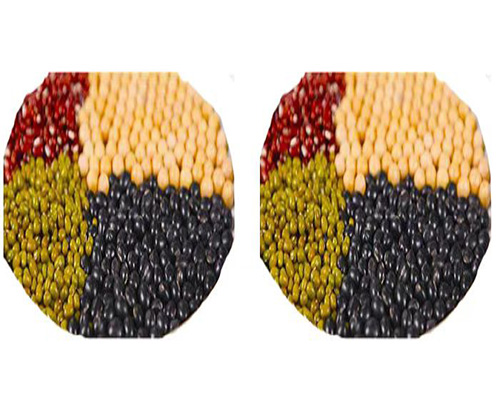

Supplement: Supplementary file 1 [file Presentation_1.zip › Pictures used in the formal experiment/3.3 LS ╘╙┴╕ ╝a═╚.jpeg]

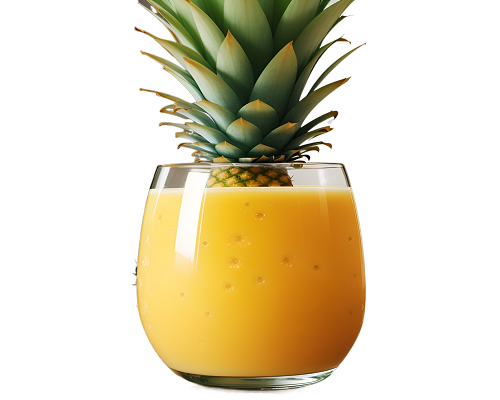

Supplement: Supplementary file 1 [file Presentation_1.zip › Pictures used in the formal experiment/30.1 FS ╣√╓¡ ▓ñ┬▄.jpeg]

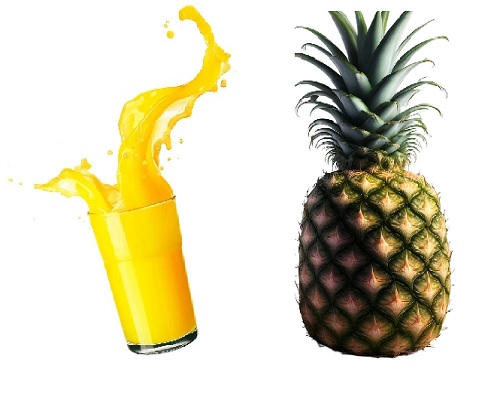

Supplement: Supplementary file 1 [file Presentation_1.zip › Pictures used in the formal experiment/30.2 JS ╣√╓¡ ▓ñ┬▄.jpeg]

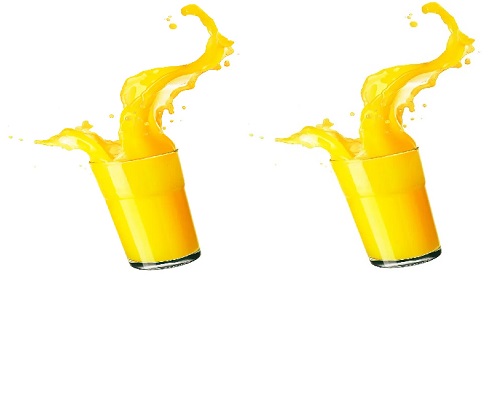

Supplement: Supplementary file 1 [file Presentation_1.zip › Pictures used in the formal experiment/30.3 LS ╣√╓¡ ▓ñ┬▄.jpeg]

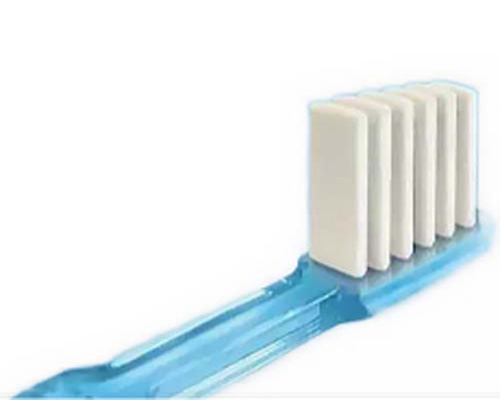

Supplement: Supplementary file 1 [file Presentation_1.zip › Pictures used in the formal experiment/4.1 FS ╤└╦ó═╖ ┐┌╧π╠╟.jpeg]

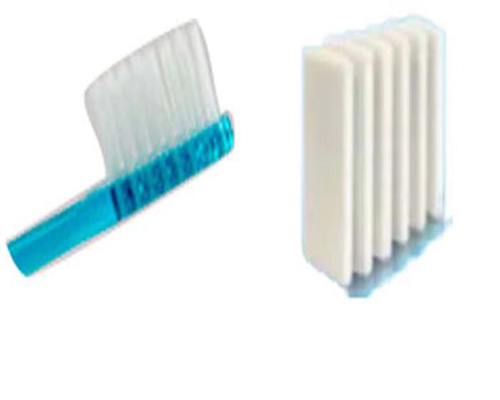

Supplement: Supplementary file 1 [file Presentation_1.zip › Pictures used in the formal experiment/4.2 JS ╤└╦ó═╖ ┐┌╧π╠╟.jpeg]

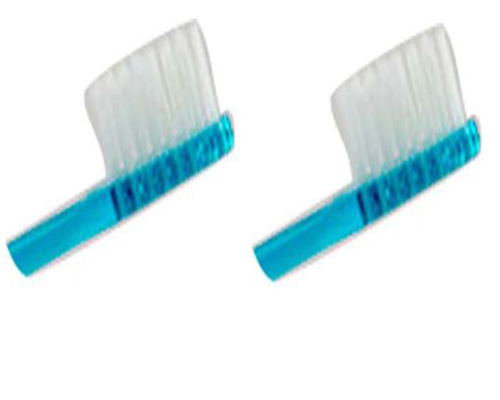

Supplement: Supplementary file 1 [file Presentation_1.zip › Pictures used in the formal experiment/4.3 LS ╤└╦ó═╖ ┐┌╧π╠╟.jpeg]

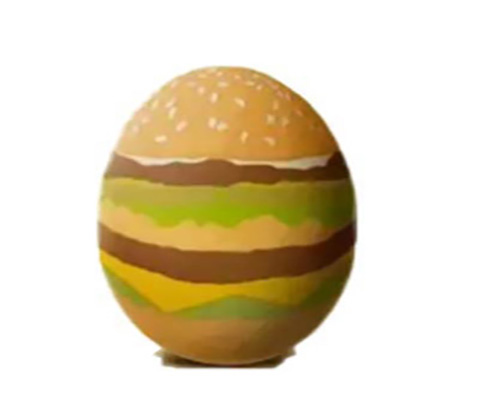

Supplement: Supplementary file 1 [file Presentation_1.zip › Pictures used in the formal experiment/5.1 FS ╝a╡░ ║║▒ñ.jpeg]

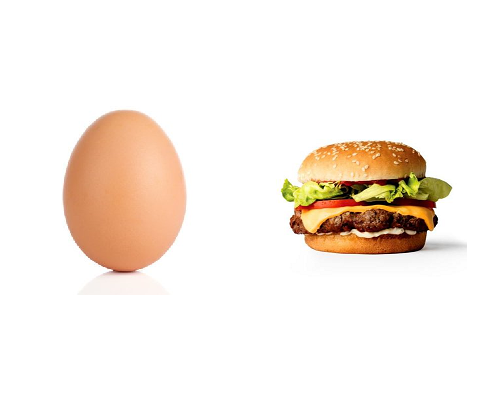

Supplement: Supplementary file 1 [file Presentation_1.zip › Pictures used in the formal experiment/5.2 JS ╝a╡░ ║║▒ñ.jpeg]

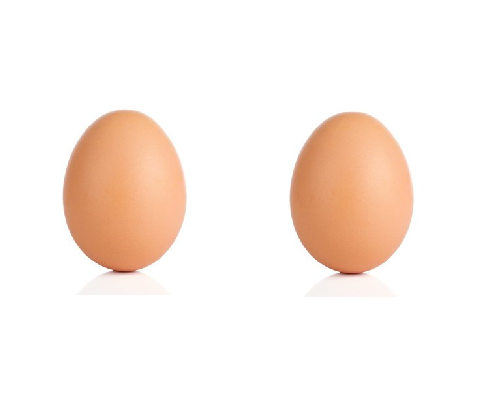

Supplement: Supplementary file 1 [file Presentation_1.zip › Pictures used in the formal experiment/5.3 LS ╝a╡░ ║║▒ñ.jpeg]

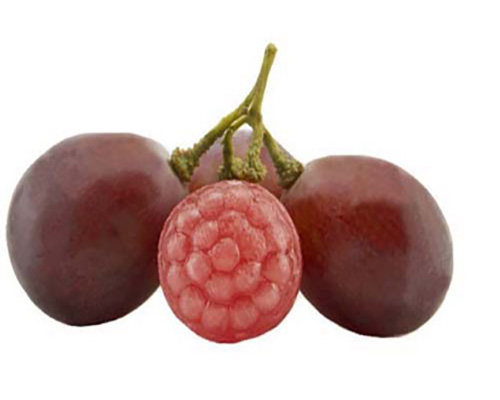

Supplement: Supplementary file 1 [file Presentation_1.zip › Pictures used in the formal experiment/6.1 FS ╞╧╠╤ ╠╟┐Θ.jpeg]

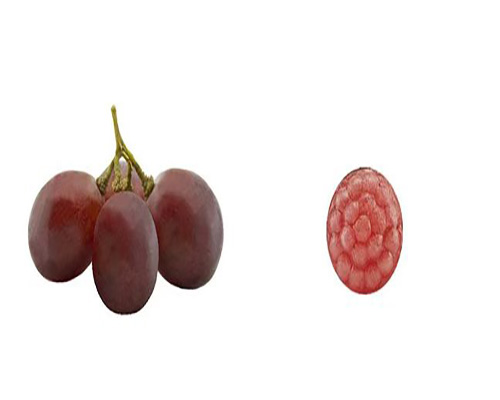

Supplement: Supplementary file 1 [file Presentation_1.zip › Pictures used in the formal experiment/6.2 JS ╞╧╠╤ ╠╟┐Θ.jpeg]

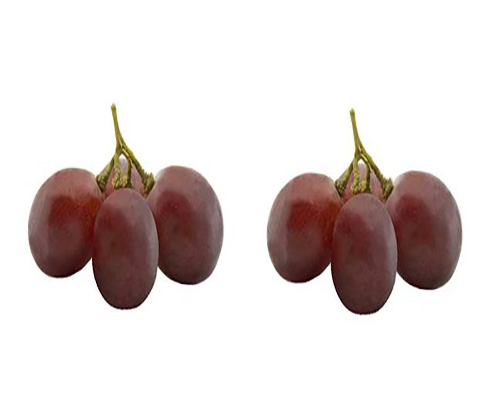

Supplement: Supplementary file 1 [file Presentation_1.zip › Pictures used in the formal experiment/6.3 LS ╞╧╠╤ ╠╟┐Θ.jpeg]

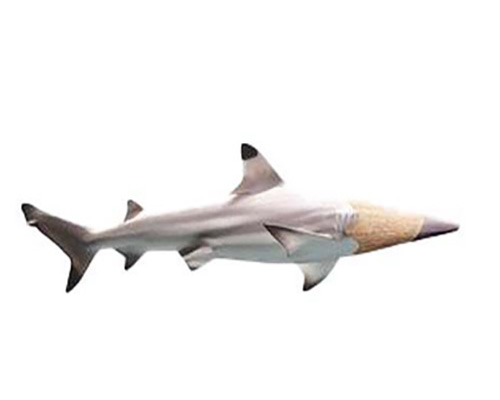

Supplement: Supplementary file 1 [file Presentation_1.zip › Pictures used in the formal experiment/7.1 FS ╟a▒╩ ÷Φ╙π.jpeg]

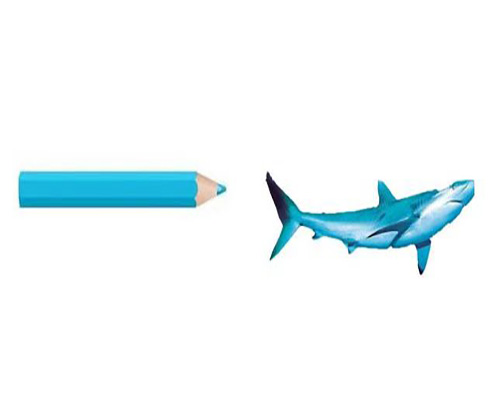

Supplement: Supplementary file 1 [file Presentation_1.zip › Pictures used in the formal experiment/7.2 JS ╟a▒╩ ÷Φ╙π.jpeg]

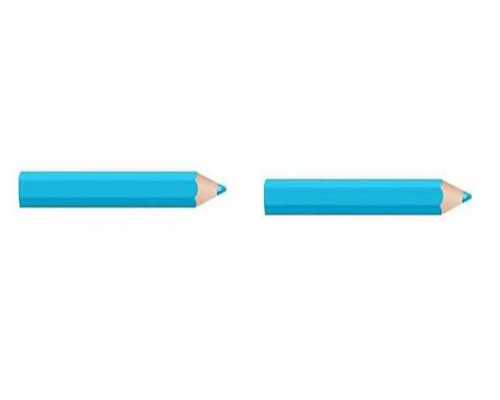

Supplement: Supplementary file 1 [file Presentation_1.zip › Pictures used in the formal experiment/7.3 LS ╟a▒╩ ÷Φ╙π.jpeg]

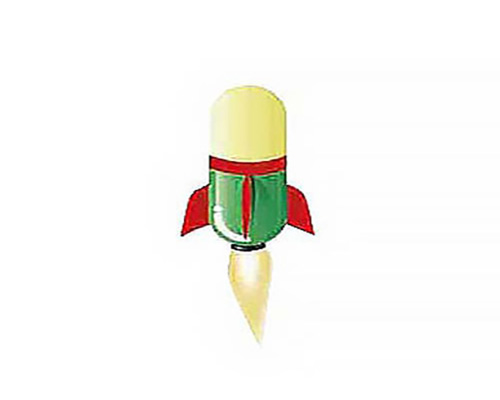

Supplement: Supplementary file 1 [file Presentation_1.zip › Pictures used in the formal experiment/8.1 FS ╗≡╝2 ╜║─╥.jpeg]

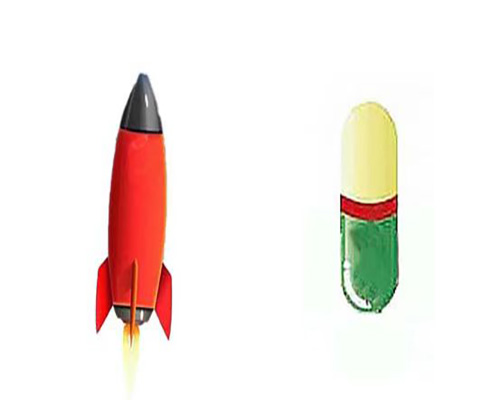

Supplement: Supplementary file 1 [file Presentation_1.zip › Pictures used in the formal experiment/8.2 JS ╗≡╝2 ╜║─╥.jpeg]

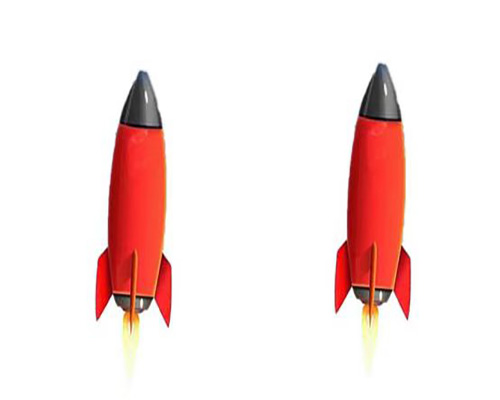

Supplement: Supplementary file 1 [file Presentation_1.zip › Pictures used in the formal experiment/8.3 LS ╗≡╝2 ╜║─╥.jpeg]

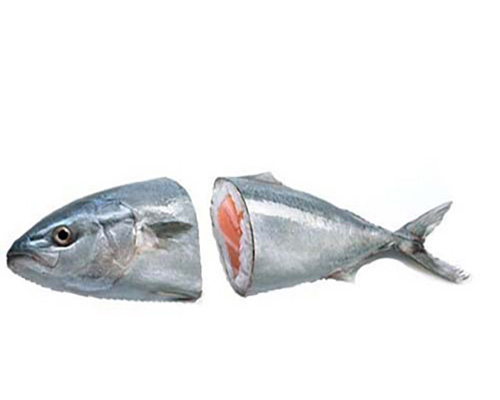

Supplement: Supplementary file 1 [file Presentation_1.zip › Pictures used in the formal experiment/9.1 FS ╧╩╙π ╩┘╦╛.jpeg]

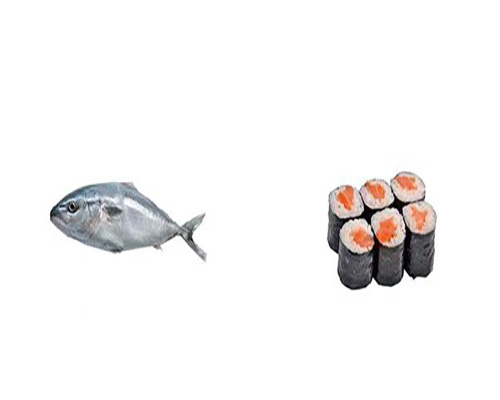

Supplement: Supplementary file 1 [file Presentation_1.zip › Pictures used in the formal experiment/9.2 JS ╧╩╙π ╩┘╦╛.jpeg]

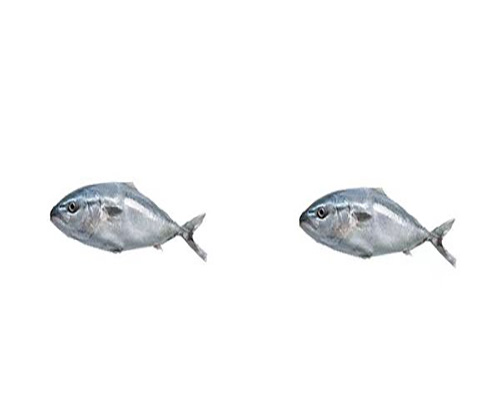

Supplement: Supplementary file 1 [file Presentation_1.zip › Pictures used in the formal experiment/9.3 LS ╧╩╙π ╩┘╦╛.jpeg]

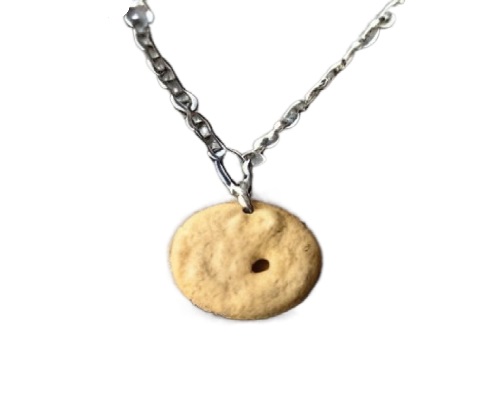

Supplement: Supplementary file 2 [file Presentation_2.zip › 31.1 FS 项链 饼干.jpeg]

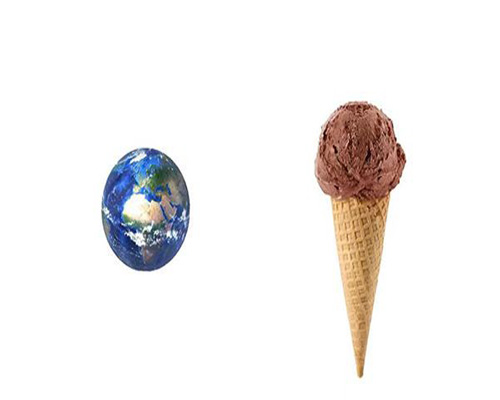

Supplement: Supplementary file 2 [file Presentation_2.zip › 32.2 JS 地球 甜筒.jpeg]

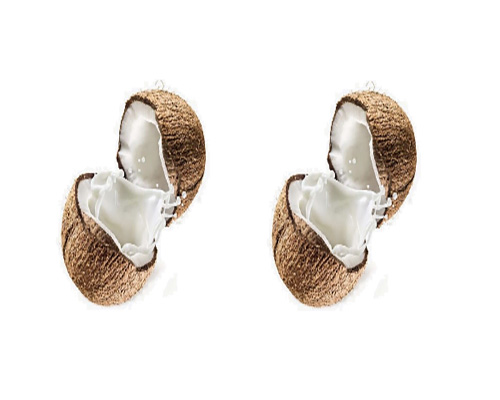

Supplement: Supplementary file 2 [file Presentation_2.zip › 33.3 LS 椰子 冰棍.jpeg]

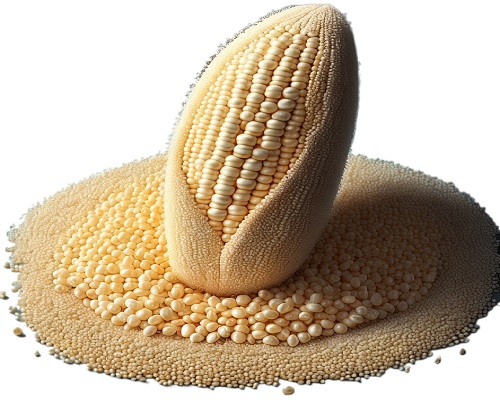

Supplement: Supplementary file 2 [file Presentation_2.zip › 34.1 FS 玉米 珠宝.jpeg]

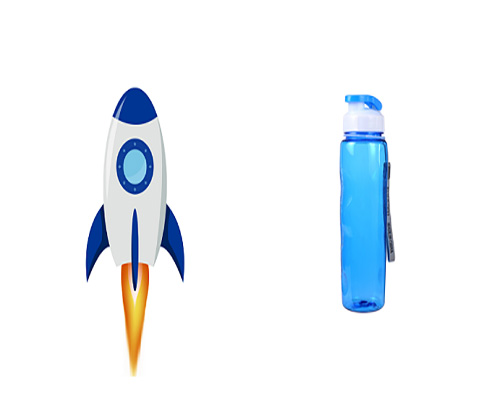

Supplement: Supplementary file 2 [file Presentation_2.zip › 35.2 JS 火箭 水杯.jpeg]

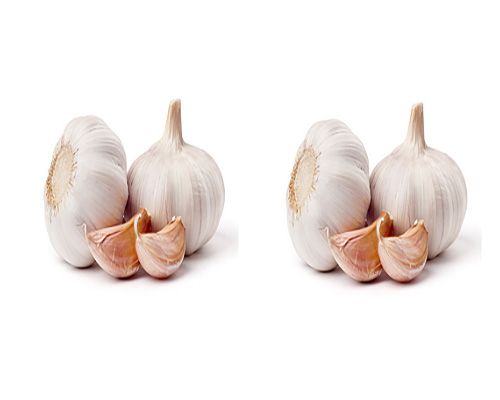

Supplement: Supplementary file 2 [file Presentation_2.zip › 36.3 LS 大蒜 橘子.jpeg]

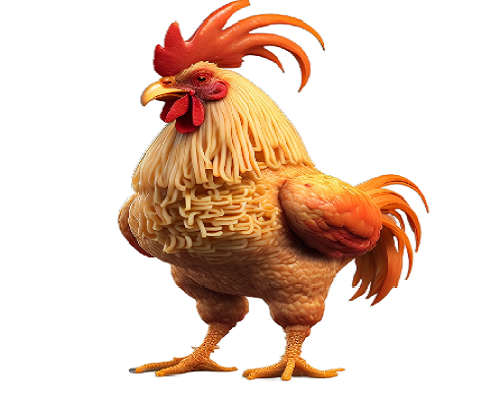

Supplement: Supplementary file 2 [file Presentation_2.zip › 37.1 FS 泡面 公鸡.jpeg]

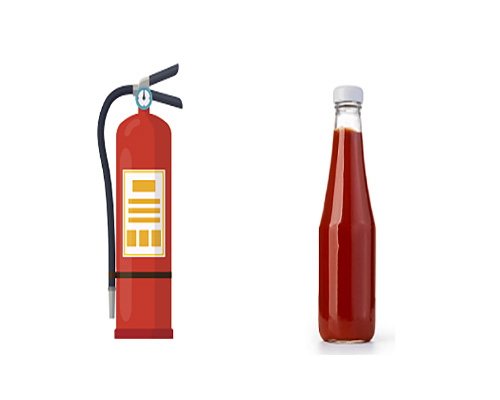

Supplement: Supplementary file 2 [file Presentation_2.zip › 38.2 JS 灭火器 辣椒酱.jpeg]

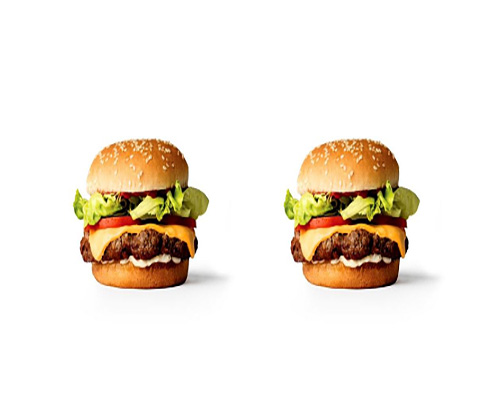

Supplement: Supplementary file 2 [file Presentation_2.zip › 39.3 LS 汉堡 碟子.jpeg]

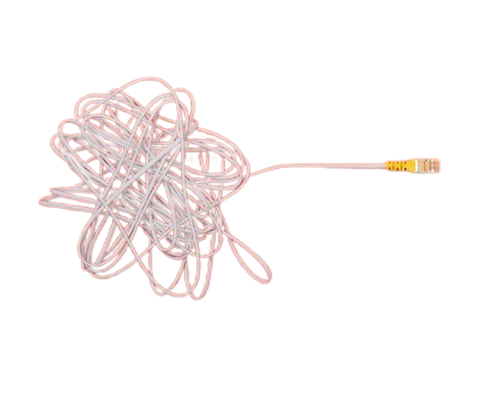

Supplement: Supplementary file 2 [file Presentation_2.zip › 40.1 FS 光缆 线团.jpeg]
