# Supplementary figures and images for: Matching different-structured advertising pictorial metaphors with verbalization forms: incongruity-based evoked response potentials evidence (part 2 of 2)
Source: Front Psychol. 2023 May 16;14:1131387. doi: 10.3389/fpsyg.2023.1131387 (PMC10227512; doi:10.3389/fpsyg.2023.1131387)

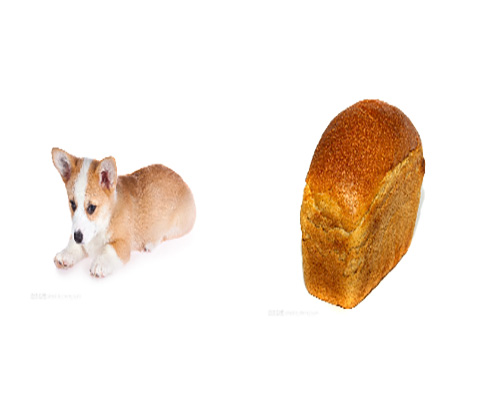

Supplement: Supplementary file 2 [file Presentation_2.zip › 41.2 JS 小狗 面包.jpeg]
